# A novel principal component based method for identifying differentially methylated regions in Illumina Infinium MethylationEPIC BeadChip data

**DOI:** 10.1080/15592294.2023.2207959

**Published:** 2023-05-17

**Authors:** Yuanchao Zheng, Kathryn L. Lunetta, Chunyu Liu, Alicia K. Smith, Richard Sherva, Mark W. Miller, Mark W. Logue

**Affiliations:** a National Center for PTSD, VA Boston Healthcare System, Boston, MA, USA; b Department of Biostatistics, Boston University School of Public Health, Boston, MA, USA; c Department of Gynecology and Obstetrics, Emory University, Atlanta, GA, USA; d Department of Psychiatry and Behavioral Sciences, Emory University School of Medicine, Atlanta, GA, USA; e Department of Psychiatry, Boston University School of Medicine, Boston, MA, USA; f Biomedical Genetics, Boston University School of Medicine, Boston, MA, USA

**Keywords:** Differentially methylated region, false positive rate, principal components

## Abstract

Differentially methylated regions (DMRs) are genomic regions with methylation patterns across multiple CpG sites that are associated with a phenotype. In this study, we proposed a Principal Component (PC) based DMR analysis method for use with data generated using the Illumina Infinium MethylationEPIC BeadChip (EPIC) array. We obtained methylation residuals by regressing the M-values of CpGs within a region on covariates, extracted PCs of the residuals, and then combined association information across PCs to obtain regional significance. Simulation-based genome-wide false positive (GFP) rates and true positive rates were estimated under a variety of conditions before determining the final version of our method, which we have named DMR_PC_. Then, DMR_PC_ and another DMR method, coMethDMR, were used to perform epigenome-wide analyses of several phenotypes known to have multiple associated methylation loci (age, sex, and smoking) in a discovery and a replication cohort. Among regions that were analysed by both methods, DMR_PC_ identified 50% more genome-wide significant age-associated DMRs than coMethDMR. The replication rate for the loci that were identified by only DMR_PC_ was higher than the rate for those that were identified by only coMethDMR (90% for DMRPC vs. 76% for coMethDMR). Furthermore, DMR_PC_ identified replicable associations in regions of moderate between-CpG correlation which are typically not analysed by coMethDMR. For the analyses of sex and smoking, the advantage of DMR_PC_ was less clear. In conclusion, DMR_PC_ is a new powerful DMR discovery tool that retains power in genomic regions with moderate correlation across CpGs.

## 1. Introduction

DNA methylation is an epigenetic (not encoded in the DNA sequence) mechanism involving the addition of a methyl group to a DNA molecule, usually at CpG sites in mammalian genomes [1]. In humans, DNA methylation has been implicated in multiple diseases, such as cancer [2] and Alzheimer’s disease [3,4]. Relatively inexpensive array-based methods for assessing genome-wide methylation have contributed to the proliferation of epigenome-wide association studies (EWASs). The Illumina Infinium MethylationEPIC BeadChip (EPIC) array [5,6] measures methylation at approximately 850,000 sites throughout the genome. This replaced the discontinued Infinium HumanMethylation450 BeadChip. Differentially methylated regions (DMRs) are genomic regions with methylation patterns across multiple CpG sites that associate with a phenotype, which are often performed as follow-up analyses after EWASs evaluating individual CpG associations. Methylation at nearby sites tends to be correlated, therefore it may be more powerful to study sets of sites to detect methylation differences [7–9].

Many statistical methods have been developed to identify DMRs. In an earlier study [10], we compared and evaluated five commonly used DMR-analysis methods developed for use with methylation-array data: comb-p [11], Bumphunter [12], DMRcate [13], mCSEA [14], and coMethDMR [15]. This 2022 study emphasized the importance of assessing genome-wide false positive (GFP) rates using genome-wide null simulations, as many of the methods had elevated false positive rates when examining genome-wide data using the parameter settings as recommended by their developers. When analysing EPIC data, coMethDMR was the only method that maintained appropriate GFP rates, although a normalizing transformation was suggested for skewed-continuous phenotypes. This led us to conclude that additional reliable methods for DMR analysis are warranted.

The current study proposes a novel Principal Component Analysis (PCA [16]; based DMR method (DMR_PC_), which we developed for the analysis of data generated using EPIC chips. It is an unsupervised analysis method, i.e., the genomic regions analysed were defined by grouping CpGs based on array annotations rather than by grouping CpGs based on the results of an analysis with a particular phenotype [17]. PCA is a popular tool that summarizes the dominant patterns of data and generates principal components (PCs) for further analyses. PCs are denoted and ordered by the percentages of the total amount of variation they explain, where the first PC (PC1) captures the most variation in the data and the second PC (PC2) explains the next greatest amount of variation, etc.

Several studies have examined PCA methods for summarizing variation in methylation data on a genome-wide level. In a study conducted by Farre et al., the authors adapted PCA to visualize and compare genome-wide patterns of DNA methylation in brain tissue and whole blood [18]. They found that PCA robustly identified DNA methylation patterns associated with certain biological factors such as age. PCA can also be implemented to summarize the correlation structure of methylation data within a genomic region. In particular, Zhang et al. [9] compared the performance of several DMR analysis methods based on PCA of a CpG set. They evaluated both a PCA analysis method which generated PCs using all available CpG sites in a set, and a method they dub Supervised Principal Component Analysis (SPCA [19; 20]; which used PCA to summarize methylation patterns for CpGs that were strongly correlated with the outcome. When analysing all CpGs in a region. Zhang et al. used the first k PCs of that captured at least 80% of the total variance in the methylation data in the region and applied a k-df likelihood-ratio test to compute the significance of the CpG set. Both their PCA and SPCA methods had well-controlled type I error rates, while SPCA was recommended when the correlation among CpG sites was strong. While the Zhang et al. study had several limitations, and the authors did not provide an implementation that could be used at scale for EWAS studies, their study demonstrated the feasibility of using PC-based methods to represent the correlation structure of DNA methylation data in a region for the purposes of testing association between a set of CpGs and a phenotype.

In this study, we propose a novel unsupervised DMR method based on PCA. First, we evaluated two alternative methods of combining information from multiple PCs: a multivariate regression method and a meta-analysis method, which we dub MultiPC and MetaPC respectively. We conducted genome-wide null simulations to evaluate GFP rate, and also performed power simulations to evaluate true positive (TP) rates using coMethDMR as a comparison. We did not evaluate many other methods for DMR analysis of methylation array data, based on our study we noted above indicating that most had inadequate GFP rate control [10]. We chose the best performing of our two PC-based methods as the final implemented method, denoted as DMR_PC_. Additionally, the ability of DMR_PC_ to identify replicable DMRs was evaluated in analyses of age, sex, and smoking using two ‘real-world’ datasets, with the performance of coMethDMR as a benchmark.

## 2. Methods

### 2.1. Definition of regions

Our method begins by separating autosomal CpGs into regions using the same database of EPIC chip genomic positions as coMethDMR. Raw genomic regions are defined using the combination of two approaches: clustering CpG sites by region type and by distance. Like coMethDMR, we first grouped CpG sites into genic (annotated to genes) and intergenic sets which are analysed separately, and CpG sites within these sets are clustered by distance. Neighboring CpG sites with no more than 200 bp between them are grouped into a region, and only regions with at least three CpG sites are retained.

### 2.2. Computing PCs of methylation residuals

Assume we have a sample of *n* subjects. For the *i*^th^ genomic region, let $CpG_{1}$, $CpG_{2}$, … , $CpG_{p}$ denote the n×1 vectors of M-values for *n* subjects of p CpG sites in the region, where p can vary for different regions. Both M-values and beta-values can be used to measure methylation levels. The beta-values are indicative of the proportion of methylated DNA at a particular site on the genome ranging from 0 to 1. M-values are logit transformed beta-values using a logarithm base of 2. M-values have been suggested to conduct differential methylation analysis, while beta-values are robust to the type of methylation quantification and have a straightforward biological interpretation [21,22]. For our method, we first remove the effects of the covariates by regressing the M-value of each probe on covariate sets (M-values ~ covariates) using linear regression models:

$${\hat{CpG}}_{1}={\hat{\alpha }}_{1}+{\hat{\beta }}_{1}^{\prime }X$$

$${\hat{CpG}}_{2}={\hat{\alpha }}_{2}+{\hat{\beta }}_{2}^{\prime }X$$

…

$${\hat{CpG}}_{p}={\hat{\alpha }}_{p}+{\hat{\beta }}_{p}^{\prime }X$$

where $X=\left(X_{1},\ldots ,X_{m}\right)$ denotes the sets of m covariates for n subjects and $X_{1}$ denotes the 1st covariate, etc. Then we compute methylation residuals as $CpG_{j}-{\hat{CpG}}_{j}$, where j=1,…,p, which are then standardized (mean of 0 and a standard deviation [SD] of 1). For numerical stability, if there is no variance of M-values at a CpG site, we add a small amount of noise using R [23] function *jitter*(). PCA is applied to standardized methylation residuals to extract p uncorrelated methylation features. In the present study, the R function *prcomp()* was used to perform PCA.

### 2.3. Analysis of PCs

We proposed two methods to compute the significance of the region by combining information across PCs: multivariate regression (MultiPC) and meta-analysis (MetaPC). These are described in detail below. When analysing multiple regions, false discovery rate (FDR) adjusted p-values (P_FDR_), also called q-values, were calculated to account for multiple testing [24].

MultiPC:

Assume the top k PCs can explain the pre-specified cumulative amount of site variation in a specific region. In the MultiPC method, we regress the phenotype on the top k PCs in a single model$\;(Phenotype\text{\textbackslash{}\textasciitilde{}}PC_{1}+\ldots +PC_{k}$). Linear regression models are used for analysis of continuous phenotypes, and generalized linear models with a logit link function are used for analysing dichotomous phenotypes. Regional significance is determined by comparing the nested model to the full model: that is, comparing the naive intercept-only model to the model including k PCs. To be specific, we used an F-test for the linear regression models and a Chi-squared test for the generalized linear model with a logit link function.

MetaPC:

In the MetaPC method, we first linearly regress each of the top k PCs on the phenotype individually ($PC_{1}\text{\textbackslash{}\textasciitilde{}}Phenotype;\;\ldots ;PC_{k}\text{\textbackslash{}\textasciitilde{}}Phenotype$). Then, those p-values are then meta-analysed, and the combined p-value is then reported as the regional significance. Two commonly used meta-analysis approaches to combine p-values were evaluated: Fisher’s method [25] and Stouffer’s method [26] as implemented in the R package *metap* [27]. If only PC1 remains in a region after filtering PCs with the pre-specified variation cut-off (k = 1), the p-value of regressing PC1 on phenotype is taken as the regional significance without applying a meta-analysis.

### 2.4. An existing DMR method: coMethdmr

CoMethDMR is an unsupervised DMR analysis method originally designed for continuous phenotypes. As noted above, coMethDMR starts with the same sets of genic and intergenic regions as we are using in MetaPC and MultiPC and similar criteria for distance between probes in its region definition. However, coMethDMR further divides these regions into subsets of correlated CpG sites using a minimal requirement of leave-one-out correlation statistics, denoted as rDrop, and filters out CpGs with rDrop less than a pre-specified threshold. In this study, when evaluating coMethDMR performance, we use an rDrop threshold of 0.4 as recommended by the coMethDMR authors. Thus, coMethDMR may divide regions into multiple smaller subregions or completely drop them from consideration if not enough CpGs pass the rDrop threshold. Then, coMethDMR uses a random coefficient mixed model to test groups of CpGs against a continuous phenotype and reports FDR-corrected p-values to account for multiple testing. The coMethDMR method is implemented in the R package *CoMethDMR* [28].

Though the grouping of CpGs into regions is similar for our method and coMethDMR, our PC-based methods do not then further drop individual probes within regions based on correlation patterns. Because PCs have the ability to summarize patterns in correlated data and prioritize the most relevant features, we hypothesized that there would be no need to drop less-correlated probes. Distinct clusters of correlated probes would presumably be represented by the individual PCs and hence the association between the probes and the phenotype would still be observed, as long as the method for combining the information across multiple PCs was efficient. However, we still required some minimal level of correlation to be represented in a region, if even just between two probes. Therefore, we have implemented a threshold for analysis based on the maximum absolute pairwise correlation (MAC). Regions with low MACs are not included in the DMR analysis as, without even a single pair of weakly correlated CpGs within those regions, individual-CpG analysis is likely the best method for testing association.

## 3. Evaluation methods

### 3.1. Study populations and covariates

In this study, two data sets were used: a Discovery and a Replication cohort. The Discovery cohort was the Translational Research Center for TBI and Stress Disorders (TRACTS) cohort. TRACTS followed a PTSD-consortium pipeline for quality control [29,30]. A detailed description of TRACTS methylation data pre-processing and QC can be found in [10]. Briefly, whole-blood methylation was assessed for 541 TRACTS cohort participants using EPIC chips. 801,812 autosomal CpG sites passed QC filters. There were 13 subjects dropped due to missing covariates and/or genotype data, which left 528 subjects for analysis. The TRACTS genotype data QC has been described in detail elsewhere [31], and was used here to compute ancestry PCs used as covariates. The Replication cohort was the National Center for PTSD (NCPTSD) cohort, which included *n* = 654 veterans and their intimate partners. The same consortium pipeline was used for QC [29,30]. Methylation was measured from whole blood using EPIC chips, and 802,682 autosomal CpG sites passed QC filters. Details of the generation of Replication cohort genotype data (used in ancestry PC calculation) are presented in [32]. Seven subjects were dropped due to missing covariates and/or genotype data, which left 647 subjects for analysis.

The Discovery cohort was used in simulations to examine GFP rates for our PC-based methods. To maintain the correlation among covariates and among CpG sites, we used simulated phenotypes and real methylation array and covariate data. This Discovery cohort was also used in power simulations to compute true positive (TP) rates using selected genomic regions. In the ‘real data’ evaluation, we used both the Discovery and Replication data sets and observed (not simulated) phenotypes.

In the Discovery and Replication cohorts, M-values for each probe were residualized for age, sex, three ancestry principal components (ancestry PC1-ancestry PC3), estimated whole blood cell proportions (CD4+ and CD8+ T cells, natural killer cells, B cells, monocytes), and smoking scores. In both cohorts, the blood cell proportions were estimated from the methylation data using the R package *minfi* [33,34]. Smoking scores were generated based on the top 39 probes from a smoking EWAS [35] as described in [36].

### 3.2. Simulation studies

We performed several simulation studies to compare the performance of MultiPC and MetaPC using different parameter settings: 1) a modest sample size null simulation (*n* = 100), 2) a large sample null simulation (*n* = 528), and 3) a ‘power’ simulation to evaluate the detection rates for simulated true loci using coMethDMR performance as a benchmark. The best-performing method based on these simulation results was implemented as DMR_PC_.

### 3.3. Parameters examined in simulation studies

We examined the effect of varying the threshold used to determine the number of PCs analysed. In particular, we analysed the top k PCs that explained at least 99%, 95%, 90%, and 80% of the variance in the region’s CpGs. We set k to be no bigger than 10 to prevent overfitting and numerical instability. Additionally, we evaluated the performance of analysing only the first PC (k=1) in MetaPC (denoted as MetaPC1) and in MultiPC (denoted as MultiPC1). In the null simulations, we also examined the impact of varying the threshold on the minimal level of correlation required for analysis, varying the MAC threshold from 0 to 0.5 by increments of 0.1.

### 3.4. Simulation Study 1 – measuring GFP rates in a sample of *n* = 100 subjects

For each of 1000 replicates, we randomly selected 100 subjects from the Discovery cohort. For these subjects, we randomly (without respect to the sample) simulated each of four phenotypes: 1) a continuous phenotype from the standard normal distribution (normal phenotype); 2) a skewed continuous phenotype from a Chi-squared one degree of freedom distribution; 3) a dichotomous phenotype with 50% cases; and 4) a dichotomous phenotype with 25% cases. For a corresponding examination of the GFP rates of coMethDMR, see our prior publication [10]. Example genome-wide null simulation code is available on GitHub (https://github.com/ggzheng/DMR_NullSimulations).

### 3.5. Simulation Study 2 – measuring GFP rates in a large sample of *n* = 528 subjects

In the second simulation study, we used all available subjects in the Discovery cohort (*n* = 528). We simulated 1000 replicates of the same four types of phenotypes as in Simulation Study 1. In Simulation Study 2, PCs of methylation residuals were computed once and remained the same across the 1000 replicates for each simulated phenotype.

### 3.6. Simulation Study 3 – evaluation of power by measuring true positive rates

To evaluate the MetaPC’s and MultiPC’s ability to identify true signals across multiple methylation correlation patterns, we picked eight representative regions for simulations, denoted as Regions 1–8. Four regions were chosen with high MAC (~0.95 and 0.99) and four with low MAC (~0.30 and 0.31) with a variable number of probes and lengths (Table 1). To generate region-specific known ‘true’ signals, we first computed the PCs for each region. To test the performance of detecting a trait that was associated with the largest portion of variability for a region, we generated a true positive signal associated with PC1 by defining our phenotype as the M-value for the probe with the highest absolute PC1 loading, which we call the ‘causal’ locus, plus random normal noise which varied by simulation replicate. We called this simulated continuous phenotype CTS_PC1_. To test performance when the phenotype was associated with one of the other primary sources of variation in the region, we created a similar phenotype from the probe with the highest factor loading from PC2 plus some random normal noise and denoted this random continuous phenotype CTS_PC2_. To examine algorithm performance when applied to a diffuse signal, we generated another continuous phenotype (denoted CTS_PC1+PC2_) by taking the mean of standardized (mean 0, SD 1) CTS_PC1_ and CTS_PC2_. In addition, we generated simulated dichotomous phenotypes from CTS_PC1_, CTS_PC2_, and CTS_PC1+PC2_ using a median cut-off, which we denoted as DTS_PC1_, DTS_PC2_ and DTS_PC1+PC2_. In each simulation, each of these six simulated variables were tested using each of the PC-based methods and parameter combination. An observed uncorrected p-value less than 0.05 was considered a TP.

**Table 1.** Summary of Representative Genomic Regions Used in Power Simulation.

Region ID	Listed Region	Length in bp	# Probes	AAC	MAC	% Variance Explained		Probe with highest absolute PC loading (causal probe)		SD added	SD added
						PC1	PC2	PC1	PC2	PC1	PC2
1	Chr6:30038712–30039600	888	33	0.62	0.98	68.21%	5.08%	cg03343571	cg22184136	7.5	2
2	Chr6:31125920–31126373	453	17	0.054	0.31	10.48%	8.19%	cg01190171	cg15724113	2	4.5
3	Chr7:27183133–27184737	1604	36	0.75	0.95	77.77%	3.03%	cg17569124	cg03744763	4.5	2.5
4	Chr1:248100585–248100614	29	4	0.96	0.99	96.85%	1.88%	cg20507276	cg00785941	15	12
5	Chr19:8117875–8117966	91	3	0.45	0.95	67.30%	30.93%	cg11245297	cg21743830	5	1.5
6	Chr10:8095121–8096372	1251	31	0.085	0.31	10.81%	4.76%	cg09728012	cg23943136	4	3
7	Chr16:67312928–67313043	115	7	0.094	0.30	23.64%	15.38%	cg06297958	cg07498606	1.5	7
8	Chr15:72104228–72104417	189	4	0.13	0.30	30.06%	27.21%	cg06546820	cg13703253	2	5

Note: *MAC: maximum absolute pairwise correlation; AAC: average absolute pairwise correlation; SD added: the standard deviation of the random noise added to the methylation (M-value) at the ‘causal’ probe.

Note, in order to ensure that there was variability in the results, so that each region was not detectable at 100% frequency or 0% frequency across methods, the standard deviation of the random normal noise added to generate CTS_PC1_, CTS_PC2_, and CTS_PC1+PC2_ was varied for the different region/phenotype combinations (See Table 1 for details). The variability added in each case was substantial so that the correlations between the resulting phenotypes and the ‘true’ causal loci were modest. The median correlations were 0.13–0.16 between CTS_PC1_ and the PC1 causal locus across regions, 0.090–0.15 between CTS_PC2_ and the PC2 causal locus across regions, and 0.11–0.19 for CTS_PC1+PC2_ and the mean of the PC1 and PC2 causal loci across regions. However, one consequence of allowing the standard deviation of the random noise to vary across phenotype and region is that the results can only be interpreted within each phenotype/region. That is, these different analyses can only be used to determine whether the relative performance of the methods is consistent across multiple different generating conditions and cannot be used to determine which kind of phenotype is easiest to detect overall.

For comparison, we also applied coMethDMR to the power simulation data. While the same regions are used as the starting point for the coMethDMR analysis, coMethDMR additionally drops less-correlated probes, which can either cause regions as analysed by MetaPC/MultiPC to be split into subregions or excluded from calculation entirely. No probes were dropped by coMethDMR in regions 3 and 4. Regions 2 and 5–8 did not fulfill coMethDMR’s criterion and were not analysed by coMethDMR. The coMethDMR analysis divided region 1 into 3 subregions with 4, 12, and 11 probes respectively. When calculating the coMethDMR TP rate for region 1, a nominally significant association in one or more of the subregions was counted as a TP.

**Figure 1.**
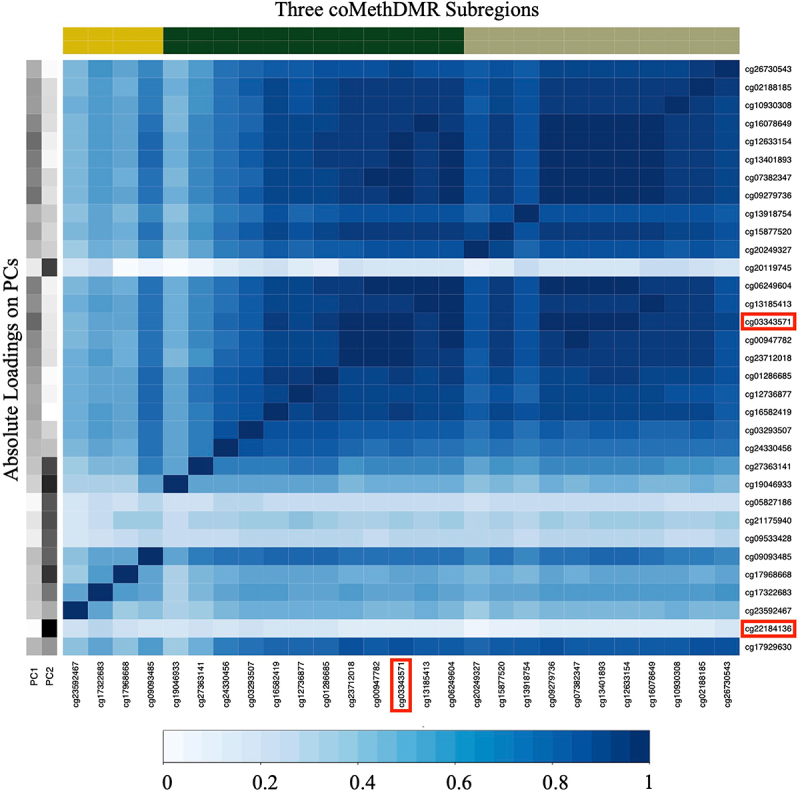
Absolute Correlations between probes in Region 1.

Note: *Region 1: Chr6:30038712-30039600.

To illustrate how the simulation variables were generated, we have included Figure 1 which represents the correlation in Region 1 as well as the absolute PC1/PC2 loadings on each CpG in that region. The two loci with the highest loadings for PC1 and PC2 were cg03343571 and cg22184136 respectively, and hence these two loci are the causal loci for the simulated variables. The probes corresponding to the three subregions as evaluated by coMethDMR are also represented.

*PC1, PC2: absolute values of PC loadings on PC1 and PC2.

*cg03343571 and cg22184136 were the most loaded probes on PC1 and PC2, where cg22184136 was dropped out by coMethDMR.

### 3.7. Application to real data

The best-performing PC-based method based on GFP and TP rates in the simulations was selected as the final proposed method, denoted as DMR_PC_. We then compared the results of DMR_PC_ and coMethDMR by applying both methods to real phenotypes using the Discovery cohort (*n* = 528) and the Replication cohort (*n* = 647). Age, sex, and smoking were chosen for examination as these are well-known to be associated with methylation at many loci [37–39]. In the Replication cohort, participants who reported never smoking, prior smokers, and current smokers were analysed as a continuous phenotype with values of 0, 1, and 2 respectively based on the reversion of methylation at many smoking-associated loci after cessation (see e.g [35]). In the Discovery cohort, smoking was analysed as a dichotomous phenotype indicating current smoking status (yes/no), as prior smoking behaviour was not assessed in this cohort. The number of subjects included in each DMR analysis varied due to missing covariate values, varying between 400 ~ 528 in the Discovery cohort and 461 ~ 647 in the Replication cohort (Table 2). Note, that when performing DMR analyses for a phenotype, the phenotype was left out of the covariate set used to compute methylation residuals.

**Table 2.** Comparison of Numbers of Regions and DMRs Reported by DMR_PC_ and coMethdmr.

Cohort	Phenotype	DMR_PC_		coMethDMR				High correlation Regions (Analyzed by Both Methods)					Moderate correlation Regions (Analyzed by DMR_PC_ Only)		
		# Regions	# DMRs	# Regions	# DMRs	# Raw Regions	# Raw DMRs	# Regions	MAC Median (IQR)	# DMRs			# Regions	MAC Median (IQR)	# DMRs
										DMR_PC_	coMethDMR	Both			
Discovery	Age	19799	8961	8423	2834	8632	2923	8423	0.75 (0.63–0.85)	4252	2834	2644	11376	0.44 (0.36–0.57)	4709
	Sex	19799	1100	8423	580	8632	583	8423	0.75 (0.63–0.85)	675	580	394	11376	0.44 (0.36–0.57)	425
	Smoking (0/1)	19704	42	8289	45	8494	45	8289	0.75 (0.63–0.85)	21	45	9	11415	0.44 (0.36–0.56)	21
Replication	Age	32251	17826	13616	5803	14014	5968	13616	0.73 (0.61–0.85)	8812	5803	5683	18635	0.46 (0.38–0.56)	9014
	Sex	32251	6282	13616	2398	14014	2416	13616	0.73 (0.61–0.85)	3419	2398	1818	18635	0.46 (0.38–0.56)	2863
	Smoking (0/1/2)	31914	17	13319	6	13719	6	13319	0.74 (0.61–0.86)	14	6	3	18595	0.45 (0.38–0.56)	3

Note: IQR= interquartile range.

*Under coMethDMR, raw regions refer to genomic regions from the original output from coMethDMR including those subregions, regions refer to genomic regions with subregions combined.

We computed the total number of regions analysed and DMRs reported for each phenotype. To have comparable estimates between coMethDMR, which often divides one of the DMR_PC_ analysis regions into subregions, only one coMethDMR TP was counted even if multiple subregions were significant. To compare the concordance between the two methods, we checked DMRs among the genomic regions analysed in the Discovery cohort by both methods, as well as only by DMR_PC_. We then examined the replication rates for novel DMRs from the analysis of the Discovery cohort. By novel DMRs, we mean DMRs without any FDR-corrected genome-wide significant individual CpG associations in the Discovery cohort that were additionally only identified by one DMR method. The individual-CpG analyses were performed using the R Package *limma* [40] with the same subjects and covariate sets as in the DMR analyses.

In addition, we evaluated both methods in terms of the computational burden. We collected the cumulative running time and the peak memory allocation during the DMR analyses. All burden tests were conducted on the Boston University Shared Computing Cluster utilizing compute nodes with the same Ivybridge architecture and Intel Xeon E5-2650v2 8-core processor.

### 3.8. DMR visualization

As part of the DMR_PC_ development, we have implemented a method to visualize DMRs identified by DMR_PC_, specifically by focusing on PCs exhibiting association with the trait of interest, and ‘high-weight’ probes with an absolute PC loading greater than the median probe weight across all probes/PCs. In our DMR plots, only trait-associated PCs with (nominal p-values<0.05) and with at least two ‘high-weight’ probes were plotted. Mean methylation (beta values) are presented for groups as defined by the phenotype being analysed.

## 4. Results

### 4.1. Simulation Study 1 – measuring genome-wide false positive rates in a sample of *n* = 100 subjects

**Figure 2.**
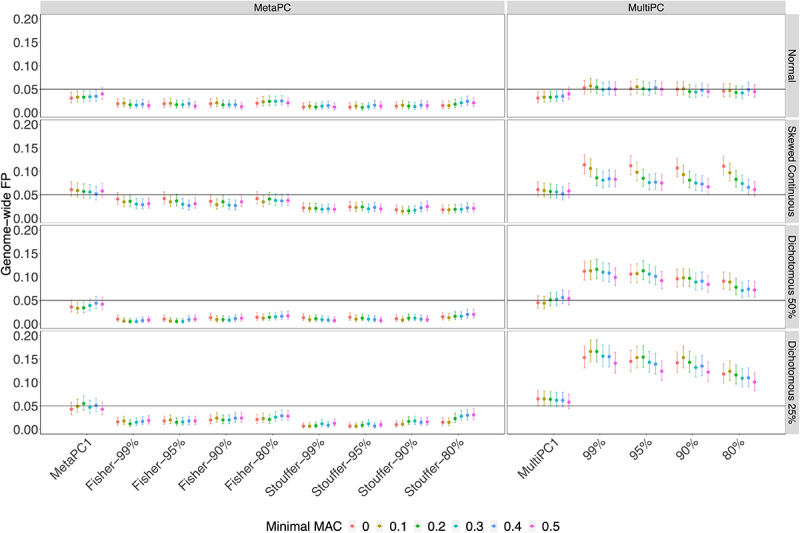
The Genome-wide False Positive Rates on Genic Regions using 100 Discovery Cohort Subjects.

Note: *Genome-wide FP: genome-wide false positive rate.

In Simulation Study 1, analyses were based on 100 randomly sampled subjects from the Discovery cohort. The genic-region results are presented in Figure 2 and Supplementary Tables 1A and 1B. MetaPC and MetaPC1 always controlled GFP rates around or under 0.05 (Supplementary Table S1A). MultiPC produced GFP rates around 0.05 when analysing the normal phenotype but slight inflation for the dichotomous and skewed continuous phenotypes across all parameter settings, with GFP rates varying between 0.071 and 0.12 for the dichotomous phenotype with 50% cases, between 0.10 and 0.17 for the dichotomous phenotype with 25% cases, and between 0.061 and 0.11 for the skewed-continuous phenotype (Supplementary Table S1B). MultiPC1 had well-controlled GFP rates around 0.05 under most conditions but had slightly inflated GFP rates on the dichotomous with 25% cases. Intergenic-region results were generally very similar to those in genic regions (Supplementary Figure S1).

*Minimal MAC:minimal maximum absolute pairwise correlation of a genomic region

*80%, 90%, 95%, 99%:minimal variance explained by PCs used.

*MetaPC:meta-analysis using multiple PCs, MultiPC: multivariate regression using multiple PCs.

*MetaPC1:meta-analysis using 1^st^ PC only, MultiPC1: multivariate regression using 1^st^ PC only.

### 4.2. Simulation Study 2 – measuring genome-wide false positive rates in a large sample of *n* = 528 subjects

**Figure 3.**
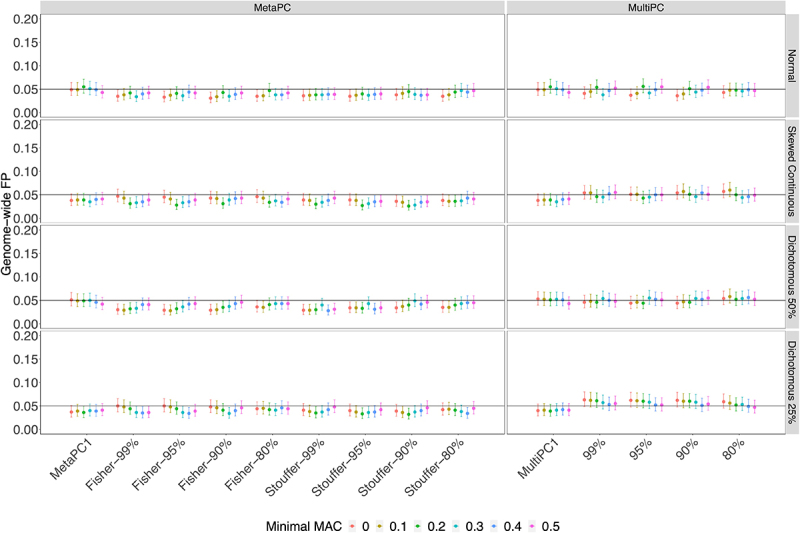
The Genome-wide False Positive Rates on Genic Regions using All Discovery Cohort Subjects (*n* = 528).

Note: *Genome-wide FP: genome-wide false positive rate.

In Simulation Study 2, we examined the genic regions using all available subjects from the Discovery cohort. All MetaPC/MetaPC1 and MultiPC/MultiPC1 methods performed well for all four phenotypes with GFP rates around 0.05 across different MAC cut-offs and the number of PCs included (Figure 3).

*Minimal MAC:minimal maximum absolute pairwise correlation of a genomic region × 80%, 90%, 95%, 99%: minimal variance explained by PCs used.

*MetaPC:meta-analysis using multiple PCs, MultiPC: multivariate regression using multiple PCs.

*MetaPC1:meta-analysis using 1st PC only, MultiPC1: multivariate regression using 1st PC only.

### 4.3. Simulation Study 3 – calculation of power by measuring true positive rates

Next, we evaluated the TP rates for simulated causal effects in eight representative regions.

**Figure 4.**
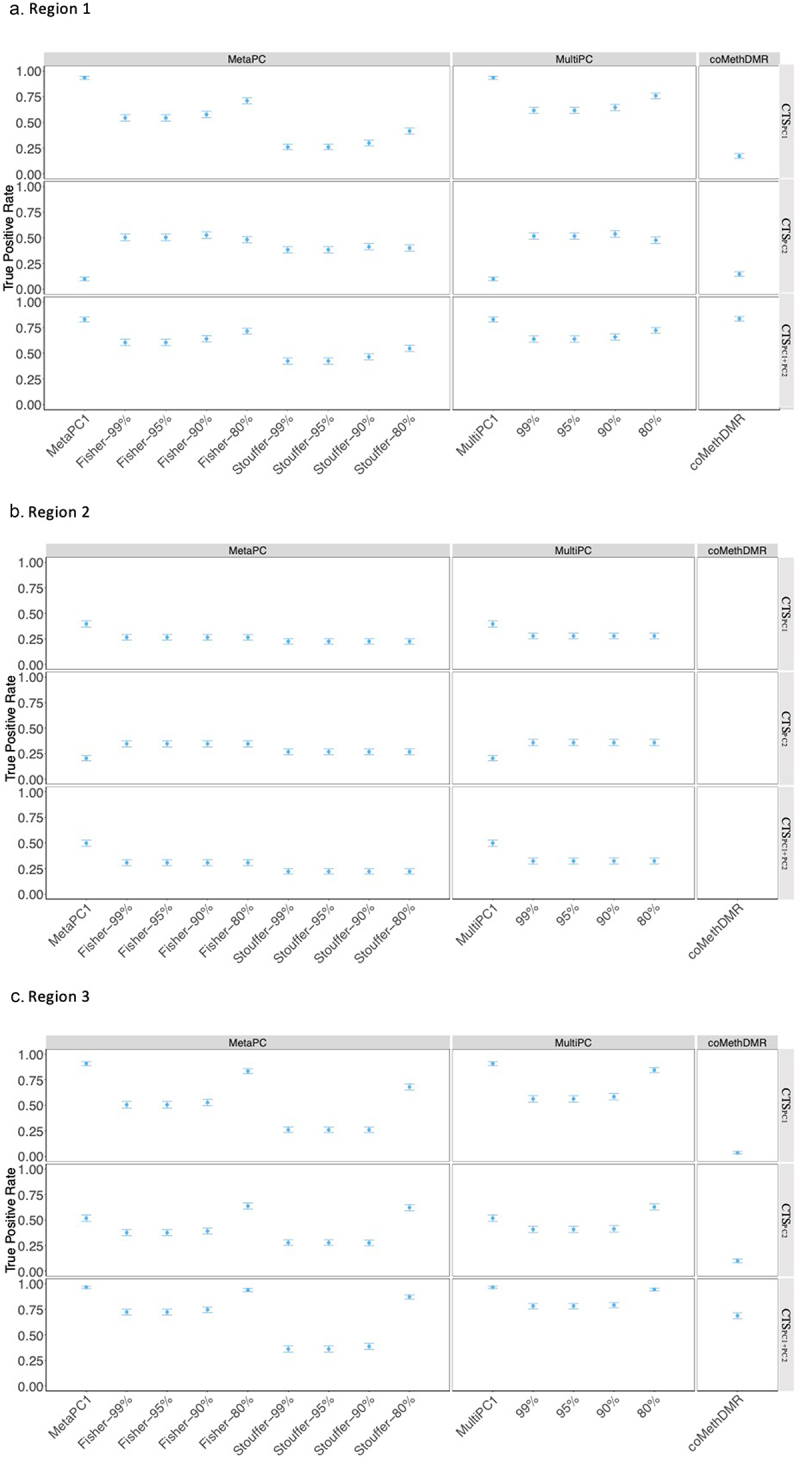
The True Positive Rates for Continuous Signals on Representative Regions: a.Region 1; b. Region 2; c. Region 3.

Note: Legend: *80%, 90%, 95%, 99%:minimal variance explained by PCs used.

Figure 4a presents the Region 1 TP rates for the different analysis methods and simulated continuous phenotypes. Unsurprisingly, MetaPC1 and MultiPC1, which only analyse the first PC, exhibited excellent performance with TP rates around 0.94 on CTS_PC1_ but had poor power with low TP rates around 0.099 to detect associations with CTS_PC2_ compared to MetaPC and MultiPC. MetaPC1/MultiPC1 also performed well on CTS_PC1+PC2_ with TP rates around 0.83. MetaPC using Fisher’s method generally performed better than MetaPC using Stouffer’s method. The best TP rates were observed with MetaPC using Fisher’s and MultiPC with a minimal 80% variance cut-off on CTS_PC1_ and CTS_PC1_+_PC2_ signals (TP rates: 0.71–0.76) or with a 90% cut-off on CTS_PC2_ (TP rates: 0.53–0.54). Next, we examined the performance of coMethDMR. The TP rates in coMethDMR were lower than MetaPC and MultiPC for CTS_PC1_ and CTS_PC2_ (TP rates: 0.17, 0.15 respectively), but higher for CTS_PC1_+_PC2_ (TP rate: 0.84) when the three subregions are considered jointly, although the true positive rates for individual subregions were lower (TP rates:0.43–0.64).

*MetaPC:meta-analysis using multiple PCs, MultiPC: multivariate regression using multiple PCs.

*MetaPC1:meta-analysis using 1st PC only, MultiPC1: multivariate regression using 1st PC only.

*CT_PC1_,CT_PC2_,CT_PC1+PC2_: continuous true positive signals simulated associated with PC1, PC2, and PC1+PC2.

In Region 2, the large number of probes required a large number of PCs to explain a sufficient amount of variation. All analyses included 10 PCs, our upper limit of the number of PCs analysed, which explained 69.40% of CpG variation. Similar to the results of Region 1, MetaPC1/MultiPC1 had higher detection rates than MetaPC and MultPC when analysing CTS_PC1_, but poorer performance when analysing CTS_PC2_ (Figure 4b). MetaPC with Fisher and MutiPC performance was similar (TP rates~0.25–0.35) and slightly higher than the performance of MetaPC with Stouffer’s method (TP rates 0.22–0.27). As previously noted, no coMethDMR results were generated for region 2.

In Region 3, MetaPC1/MultiPC1 performed well relative to the other PC-based methods when applied to CTS_PC1_ but had poorer performance when analysing CTS_PC2_. Apart from MetaPC1/MultiPC1, MetaPC using Fisher’s and MultiPC with an 80% variance cut-off had the highest TP rates (Figure 4c). Similar to the results in Region 1, coMethDMR had much lower TP rates than the PC-based methods when analysing CTS_PC1_ and CTS_PC2_, and a comparable TP rate when analysing CTS_PC1+PC2_. The pattern of associations were very similar in Regions 4–8 (Supplementary Figure S2A-2E).

Results for dichotomous phenotypes were mixed. CoMethDMR outperformed MetaPC and MultiPC when analysing DTS_PC1_ and DTS_PC1+PC2_ in Region 1 (CoMethDMR TP rates 0.66 and 0.78; MetaPC and MultiPC TP rates 0. 22–0.77). However, MetaPC using Fisher’s meta-analysis and MultiPC with an 80% variance had TP rates that were similar or higher than coMethDMR otherwise (MetaPC TP rates 0.30–0.86, coMethDMR TP rates 0.14–0.78). For a full listing of the TP rates for all phenotypes and regions, see Supplementary Table S3A-3E.

### 4.4. Summary of simulation results

Perhaps unsurprisingly, MetaPC1/MultiPC1, which focused on the first PC, were consistently the best-performing method when the causal locus was strongly weighted in the first PC, but not when analysing CTS_PC2_. Therefore, we cannot recommend MetaPC1/MultiPC1 for use, to avoid the risk of missing important signals. Apart from MetaPC1/MultiPC1, MetaPC using Fisher’s meta-analysis method with a minimal 80% variance explained cut-off outperformed MultiPC in terms of both GFP and TP rates. This best-performing PC-based method was chosen as our final method, denoted as DMR_PC_. When compared to the coMethDMR in terms of power, our chosen DMR_PC_ method had a higher TP rate for the continuous phenotypes, except when analysing CTS_PC1+PC2_ in region 1. However, that is only when we combine the signal from the three coMethDMR subregions into a single TP rate. When examining the coMethDMR results for the 3 subregions individually, they each had a lower TP rate than DMR_PC_. We also propose using a MAC cut-off at 0.3 for DMR_PC_ analysis to require that examined regions exhibit a certain minimal amount of correlation between at least two loci. This is a low correlation cut-off that did not cause any GFP inflation and also helps reduce the multiple-testing penalty.

### 4.5. Application to real data

Next, we evaluated DMR_PC_ by examining its ability to detect age, sex, and smoking-associated DMRs in genic regions, using coMethDMR performance as a baseline. Both the Discovery and Replication cohorts were analysed. DMR_PC_ examined 2.35 ~ 2.40 times the number of regions analysed by coMethDMR (Table 2), including all of the regions as analysed by coMethDMR. As expected, given the stricter inclusion criteria used by coMethDMR, the median MACs of regions analysed by both methods were around 0.73 ~ 0.75 (interquartile rage: 0.61 ~ 0.86, Range: 0.31 ~ 1.00). For brevity’s sake, we will call these regions which were analysed by both coMethDMR and DMR_PC_ the ‘high correlation regions.’ DMR_PC_ additionally analysed a batch of regions with median MAC around 0.44 ~ 0.46 (interquartile rage: 0.36 ~ 0.57, Range: 0.30 ~ 0.99), which we will call the ‘moderate correlation regions.’

In the Discovery dataset, DMR_PC_ and coMethDMR identified 8,961 and 2,834 age-associated DMRs respectively (Table 2). Among regions analysed by both methods (the high correlation regions), DMR_PC_ identified 93.30% of the DMRs identified by coMethDMR and 1,608 DMRs that were not identified by coMethDMR, where coMethDMR captured 190 DMRs that were not identified by DMR_PC_. DMR_PC_ additionally identified 4,709 DMRs in the 11,376 moderate correlation regions that weren’t analysed by CoMethDMR. In the Replication cohort, the results were similar. DMR_PC_ identified approximately 50% more DMRs than coMethDMR in the high correlation regions, including over 97% of the DMRs identified by coMethDMR. Also in the Replication cohort, DMR_PC_ identified 48.37% of the moderate correlation regions as age-associated DMRs compared to 41.39% in the Discovery dataset.

We next examined age-associated DMRs from the Discovery-cohort analysis for replication. We were particularly interested in ‘novel’ regions, where ‘novel’ implies DMRs that contained no genome-wide significant age-associated individual loci and were only uniquely identified by either DMR_PC_ or coMethDMR in the Discovery cohort. The number of genome-wide significant individual CpGs and DMRs overlapping single-CpG hits are summarized in Supplementary Tables S4 and S5. In the high correlation regions, DMR_PC_ identified 58 age-associated novel DMRs (Table 3), and 89.66% of these replicated based on genome-wide significant individual probe and/or DMR associations observed in the Replication cohort. In comparison, coMethDMR identified 29 age-associated novel DMRs and had a lower replication rate of 75.66%. In the moderate correlation regions, DMR_PC_ identified 49 novel age-related DMRs with a replication rate of 89.80%, nearly identical to the replication rate observed in the novel DMRs from the high-correlation regions.

**Table 3.** Replicate ‘Novel’ DMRs without EWAS Hits in the Discovery cohort that were only identified by DMR_PC_ or coMethdmr.

Phenotype	Novel DMRs Uniquely Identify by DMR_PC_										Novel DMRs Uniquely Identify by coMethDMR				
	High Correlation Regions					Moderate Correlation Regions					High Correlation Regions				
	# All	# with EWAS Replication	# with DMR Replication	with EWAS/DMR Replication		# All	# with EWAS Replication	# with DMR Replication	with EWAS/DMR Replication		# All	# with EWAS Replication	# with DMR Replication	with EWAS/DMR Replication	
				n	%				n	%				n	%
Age	58	51	43	52	89.66	49	44	36	44	89.80	29	22	14	22	75.86
Sex	52	32	29	32	61.54	62	41	21	41	66.13	88	63	39	64	72.73
Smoking	1	0	0	0	0	2	0	0	0	0	14	1	0	1	7.14

Next, we performed a DMR analysis of sex. There were 50 (9.47%) and 237 (36.63%) female subjects in the Discovery and Replication cohorts respectively. In the Discovery cohort, in the high correlation regions, DMR_PC_ identified 1.90 times the total number of sex-associated DMRs and 67.93% of the sex-associated DMRs reported by coMethDMR. In the Replication cohort, DMR_PC_ identified 2.62 times the total number of sex-associated DMRs and 75.81% of the sex-associated DMRs reported by coMethDMR. In the Discovery cohort, DMR_PC_ found 52 novel sex-associated DMRs with a 61.54% replication rate from the high-correlation regions and 62 novel DMRs with a 66.13% replication rate from the moderate-correlation regions. CoMethDMR identified 88 novel DMRs with a with a replication rate of 72.73%.

We next examined smoking. DMR_PC_ found 42 and 17 smoking-associated DMRs in the Discovery and Replication cohorts respectively, and coMethDMR found 45 and 6 associated DMRs in the Discovery and Replication cohorts respectively. There were only 1 and 2 smoking-related novel DMRs for DMR_PC_ from the high and moderate correlation regions, and 14 for coMethDMR from the high correlation regions. None of the 3 smoking-related novel DMRs from DMR_PC_ were replicated, and only 1 smoking-associated region from the coMethDMR analysis was replicated. We additionally examined replication using smoking EWAS results from an external study conducted by Christiansen et al. [41], which lent support for two more of the smoking DMRs identified by coMethDMR, but none of the novel DMR_PC_ DMRs.

### 4.6. Computational burden

We compared the computational burden between DMR_PC_ and coMethDMR in terms of the cumulative running time and the peak memory allocation in the analyses of age, sex, and smoking. In the Discovery dataset, DMR_PC_ and used 31.69 ~ 31.93 GB of peak memory and coMethDMR used 29.11 GB of peak memory. In terms of running time, DMR_PC_ took 7.77 ~ 9.69 hours to complete and coMethDMR took 7.06 ~ 7.76 hours to complete (Table 4). Similarly, in the Replication dataset, DMR_PC_ used 38.49 ~ 38.60 GB peak memory and .41 ~ 12.03 hours of cumulative running time and coMethDMR used 34.87 ~ 34.96 GB in peak memory, and 9 and 7.60 ~ 8.94 hours of cumulative running time.

**Table 4.** Comparison of Relative Computational Burden between DMR_PC_ and coMethdmr.

Cohort	Phenotype	# Subjects	DMR_PC_		coMethDMR	
			Peak Memory in GB	Time in hours	Peak Memory in GB	Time in hours
Discovery	Age	528	31.93	9.69	29.11	7.76
	Sex	528	31.93	9.46	29.11	7.11
	Smoking (0/1)	400	31.69	7.77	29.11	7.06
Replication	Age	647	38.60	12.03	34.87	8.94
	Sex	647	38.60	11.99	34.87	8.55
	Smoking (0,1,2)	461	38.49	9.41	34.96	7.60

Note: *Results in coMethDMR were based on two functions: CoMethAllRegions() and lmmTestAllRegions().

*Results should be interpreted relatively, as they may vary depending on the computer system used. The estimates of peak memory and running time were computed by the shared compute nodes at Boston University Shared Computing Cluster.

### 4.7. DMR visualization

**Figure 5.**
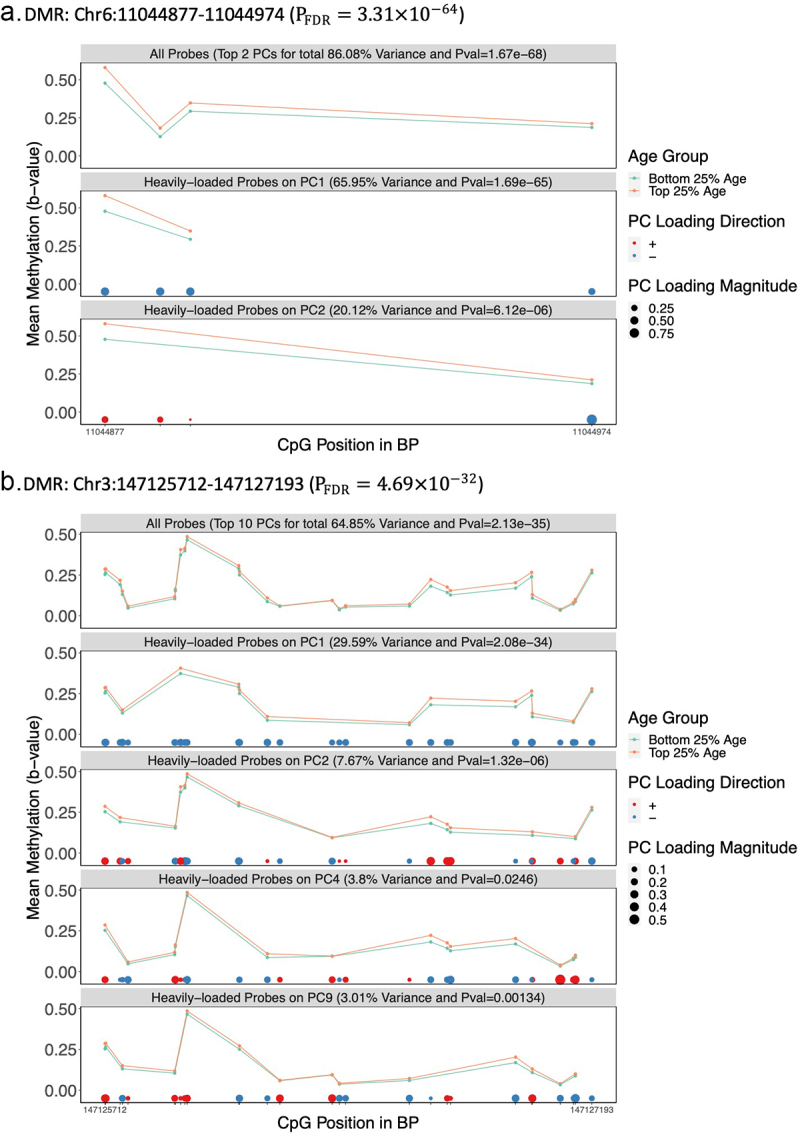
Visualization of Two Age-related DMRs in the Discovery Cohort a.DMR: Chr6:11044877 –11,044,974 ($P_{FDR}=3.31\times 10^{-64}$). b.DMR: Chr3:147125712 –147,127,193 ($P_{FDR}=4.69\times 10^{-32}$).

Note: Legend: *The DMR Chr6:11044877-11044974 was the most significant age-related DMR in the Discovery cohort $P_{FDR}=3.31\times 10^{-64}$ with 4 probes available and a MAC of 0.77. In this region, 2 PCs were adopted in the DMR_PC_ analysis to explain 86.08% total variance of methylation residuals.

As part of the DMR_PC_ implementation, we created a visualization tool for the display of DMR_PC_ results, highlighting the methylation values for the probes with high weights for trait-associated PCs. To demonstrate this tool, we compared two selected age-associated DMRs in the Discovery cohort: Chr6:11044877–11044974 (P_FDR_=$3.31\times 10^{-64}$) and Chr3:147125712–147127193 (P_FDR_=$4.69\times 10^{-32}$). The Chr 6 DMR was smaller and included 4 probes. In this region, two PCs were analysed, and PC1 explained the majority of variability across the region (65.95%) with the same direction for of all its probe loadings. The mean of methylation levels for the two age groups differed more for probes with high PC1 weights than for probes with high PC2 weights (Figure 5a). The Chr 3 region included 28 probes. PC1 also helped capture the differential methylation patterns of this region (Figure 5b). Compared to using all probes, the difference in mean methylation levels for probes with high PC1 weights differed more between age groups.

*The DMR Chr3:147125712-147127193 ($P_{FDR}=4.69\times 10^{-32}$) had 28 probes available within the region and a MAC of 0.65. In the region, 10 PCs were adopted in the DMRPC analysis to explain 64.85% total variance of methylation residuals.

*Only PCs with nominal p-values<0.05 and probes (at least 2) on each PC with absolute PC loadings above the estimated 50% quantile were plotted. Note, weight signs (±) are arbitrary in PCA.

## 5. Discussion

In this study, we developed an unsupervised PC-based method for identifying DMRs for use with EPIC methylation-chip data. We proposed using a PC-based method due to the prior work demonstrating that PCs can effectively summarize genome-wide methylation data [18] and a study demonstrating that PCs can be used to summarize variation in a region to test association with a trait [9]. However, the optimal number of PCs to be included and method of combining information from multiple PCs were not readily apparent. Therefore, we empirically compared the performance of two different methods for analysing and combining information across multiple PCs, MetaPC and MultiPC. The MetaPC method requiring PCs explaining at least 80% variance be included in the model using Fisher’s method for meta-analysis had the best performance across all parameter settings in both GFP rates and TP rates. Hence, MetaPC was then chosen as our final proposed PC-based method, which we are calling DMR_PC_.

Based on our power simulations, the TP rate for DMR_PC_ compared favourably with the performance of coMethDMR, another DMR analysis method. In simulation regions that were analysed by both DMR_PC_ and coMethDMR, the performance of DMR_PC_ was significantly better than coMethDMR when analysing simulated continuous phenotypes corresponding to the major sources of variation in the region (CTS_PC1_ and CTS_PC2_). That is, TP rates for coMethDMR when analysing these phenotypes varied from 0.034 to 0.44 while the DMR_PC_ TP rates over the same regions and phenotypes were uniformly higher and varied from 0.48 to 0.83 (Supplementary Table S3). In analyses of a third simulated continuous phenotype, representing a diffuse signal (CTS_PC1+PC2_), the TP rates for of coMethDMR and DMR_PC_ were similar; TP rates for coMethDMR varied from 0.69 to 0.92, while DMR_PC_ TP rates varied from 0.72 to 0.97, with DMR_PC_ having higher performance in two of the three regions. Therefore, we can conclude that DMR_PC_ performs better than coMethDMR when the trait analysed is continuous and corresponds to one of the major sources of variation in a region and has similar performance for continuous traits when the signal is diffuse. Of course, in practice, it is not possible to know in advance how whether a phenotype corresponds to one or more than one source of regional variation, and this may well vary from region to region. Overall, the comparison between DMR_PC_ and coMethDMR performance when applied to simulated phenotypes indicated that DMR_PC_ would, on-average, improve power when analysing continuous traits.

This was born out when we examined the results of a DMR_PC_ when applied to an analysis of age in a Discovery and a Replication cohort. Among high correlation regions that were analysed by both methods, DMR_PC_ found 50% more DMRs than coMethDMR in both cohorts and identified over 90% of the DMRs found by coMethDMR. We also performed cross-cohort validation on ‘novel’ DMRs (without any genome-wide significant individual probes from Discovery cohort and only identified uniquely by one method). DMR_PC_ identified more novel loci, and a higher proportion of the age-related novel DMR_PC_ DMRs replicated.

When examining the categorical phenotypes and semi-categorical phenotypes (smoking in the replication cohort) results were mixed. We did not observe any consistent performance advantage for DMR_PC_ relative to coMethDMR when applied to categorical phenotypes. In the power simulations, DMR_PC_ had higher TP rates in 6 of the nine categorical phenotype/region combinations. DMR_PC_ identified more sex-associated DMRs than coMethDMR in the high correlation regions, but had fewer novel DMRs, presumably due to the number of DMR_PC_ regions which were included genome-wide significant individual CpGs. DMR_PC_’s replication rate for novel sex-associated DMRs was lower than that for coMethDMR. DMR_PC_ found only 1 novel smoking-associated DMR, while coMethDMR identified 14, but with a very low replication rate of 7%, which indicates that low power may have been a complicating factor for both methods when applied to smoking.

All of the comparisons noted above are done on regions that were analysed by both DMR_PC_ and coMethDMR. However, by only requiring a moderate level of pairwise correlation in a region, DMR_PC_ analyses many more regions that aren’t assessed by coMethDMR, at least at the default DMR_PC_ and coMethDMR settings. DMR_PC_ identified 49 age-associated DMRs in moderate correlation regions in the Discovery cohort that were not identified by single-CpG analysis. The DMR_PC_ replication rate for these loci was virtually identical to the novel loci identified in high-correlation regions, indicating that they have similar reliability. In the analysis of sex, DMR_PC_ identified 41 novel associations in the moderate correlation regions, and their replication rate was even higher than that of the high correlation region sex-associated DMRs (66% vs 62%). Therefore, our analyses of sex and age support using PC-based DMR analysis in moderate correlation regions, as these regions can produce useful and reliable associations.

There are several limitations to the proposed work. First, we note that our performance evaluation was based on data that had been cleaned with a pipeline that accounted for chip and position effects. While DMR_PC_ performed well with these data, we would not necessarily expect that it would maintain appropriate type 1 error rates in data with batch effects or in analysing data that had not been appropriately balanced when assigning samples to chips/positions. Sound data cleaning and batch effect correction is still required before analysis with DMR_PC_. We have not evaluated DMR_PC_ on cohorts of less than 100 subjects due to difficulty accurately modelling the correlation structure within a region in very small cohorts. Another limitation is that the simulation examining power was only based on eight regions. However, these regions were picked to be representative of a large number of situations. Additionally, neither DMR_PC_ nor coMethDMR was able to identify many smoking-related DMRs. This may be due to low power. We also note that our method was created and validated for use with EPIC data. However, it could be adapted to Infinium HumanMethylation450 BeadChip data or whole-genome bisulphite sequence data after careful evaluation for GFP rate and power. Additionally, we only examined the performance of DMR_PC_ as applied to methylation data generated from whole-blood samples. However, while we expect that patterns of methylation data would differ based on the tissue, we note that DMR_PC_ performed well across a variety of region types, including large and small regions as well as regions with moderate correlation and high correlation, and hence, we would expect that the method would also perform well in other tissues. However, caution would suggest that genome-wide null simulations are advisable to confirm appropriate GFP control when using DMR_PC_ with new tissue/covariate set combinations. See our GitHub page, https://github.com/ggzheng/DMR_NullSimulations, for code that can be used to implement genome-wide null simulations similar to those performed here in Simulation 1 and 2 and our prior publication [10]. Another limitation is that we have only evaluated DMR_PC_’s performance on autosomal CpG sites. Further validation is needed to ensure that DMR_PC_ performs appropriately on the sex-linked chromosomes. Finally, we note that when comparing DMR_PC_ to coMethDMR performance, we only evaluated coMethDMR using the parameters as suggested by Gomez et al. in the original paper [15]. It may be possible to find parameter settings that improve coMethDMR performance relative to what we see here. However, the performance of DMR_PC_ across the simulated and real phenotypes as we have presented here supports the use of PCs to summarize regional DNA methylation data and as a tool for DMR discovery.

In summary, DMR_PC_ is a new powerful DMR analysis tool for EPIC data, allowing efficient analysis of regions with modest between-CpG correlation. DMR_PC_ takes advantage of PCA to extract PCs summarizing the dominant patterns in nearby correlated methylation loci. DMR_PC_ is robust in controlling GFP rates for phenotypes with various distributions and had similar or better performance when analysing continuous phenotypes than a competing method, coMethDMR. Both methods were similar in terms of peak memory and running time, notwithstanding the fact that DMR_PC_ examined more than twice the number of regions analysed by coMethDMR. To allow easy implementation of DMR_PC_, we have uploaded our R scripts to GitHub (https://github.com/ggzheng/DMRpc) Example code for plotting DMRs as presented in Figure 5 is also provided. We would recommend use of DMR_PC_ for the analysis of continuous phenotype data. DMR_PC_ would also be useful for the analysis of categorical phenotypes, although whether or not it has an advantage over coMethDMR on categorical phenotypes is less clear, and it may be beneficial to run both methods.

## Supplementary Material

Supplemental Material

## Data Availability

The datasets analysed during the current study are not publicly available. Qualified investigators can apply to the PTSD Genetics and TRACTS data repositories to gain access to these data via a Data Use Agreement. Please contact Dr. MW Miller regarding access to methylation data from PTSD Genetics and TRACTS data repositories.
