# Supplementary material for: A novel principal component based method for identifying differentially methylated regions in Illumina Infinium MethylationEPIC BeadChip data
Source: Epigenetics. 2023 May 17;18(1):2207959. doi: 10.1080/15592294.2023.2207959 (PMC10193914; doi:10.1080/15592294.2023.2207959)
Supplement: Supplemental Material [file KEPI_A_2207959_SM9080.docx]

**Supplementary Materials for:**

**A Novel Principal Component based Method for Identifying Differentially Methylated Regions using Illumina EPIC Methylation Arrays.**

**by Yuanchao Zheng PhD et al.**

**Supplementary Table and Figure Legends**

Supplementary Table 1. The Genome-wide False Positive Rates on Genic Regions using 100 Discovery Cohort Subjects:

1. MetaPC; B. MultiPC

Supplementary Table 2. The Number of Genomic Regions Analyzed in Genome-wide False Positive Rates using 100 Discovery Cohort Subjects

Supplementary Table 3. The Summary of True Positive Rates on Representative Regions

1. MetaPC using Fisher’s Method; B. MetaPC using Stouffer’s Method; C. MultiPC; D. coMethDMR

Supplementary Table 4. The Number of Genome-wide Significant CpG Sites in EWASs

Supplementary Figure 1. The Genome-wide False Positive Rates on Intergenic Regions using 100 Discovery Cohort Subjects

Supplementary Figure 2. The True Positive Rates for Continuous Signals on Representative Regions

**Supplementary** **Table 1.** The Genome-wide False Positive Rates on Genic Regions using 100 Discovery Cohort Subjects

1. MetaPC

| **Minimal**  **MAC** | **Phenotype** | **1st PC** | **Fisher’s Method** | | | | **Stouffer’s Method** | | | |
| --- | --- | --- | --- | --- | --- | --- | --- | --- | --- | --- |
|  |  |  | **99%** | **95%** | **90%** | **80%** | **99%** | **95%** | **90%** | **80%** |
| 0 | Normal | 0.031(0.021-0.044) | 0.019(0.011-0.03) | 0.019(0.011-0.03) | 0.019(0.011-0.03) | 0.02(0.012-0.031) | 0.012(0.0062-0.021) | 0.012(0.0062-0.021) | 0.014(0.0077-0.023) | 0.015(0.0084-0.025) |
| 0 | Skewed Continuous | 0.061(0.047-0.078) | 0.041(0.03-0.055) | 0.042(0.03-0.056) | 0.036(0.025-0.049) | 0.042(0.03-0.056) | 0.022(0.014-0.033) | 0.024(0.015-0.036) | 0.018(0.011-0.028) | 0.018(0.011-0.028) |
| 0 | Dichotomous 50% | 0.036(0.025-0.049) | 0.01(0.0048-0.018) | 0.01(0.0048-0.018) | 0.013(0.0069-0.022) | 0.014(0.0077-0.023) | 0.013(0.0069-0.022) | 0.014(0.0077-0.023) | 0.011(0.0055-0.02) | 0.015(0.0084-0.025) |
| 0 | Dichotomous 25% | 0.043(0.031-0.057) | 0.016(0.0092-0.026) | 0.018(0.011-0.028) | 0.02(0.012-0.031) | 0.021(0.013-0.032) | 0.007(0.0028-0.014) | 0.007(0.0028-0.014) | 0.01(0.0048-0.018) | 0.015(0.0084-0.025) |
| 0.1 | Normal | 0.033(0.023-0.046) | 0.02(0.012-0.031) | 0.02(0.012-0.031) | 0.021(0.013-0.032) | 0.023(0.015-0.034) | 0.014(0.0077-0.023) | 0.014(0.0077-0.023) | 0.016(0.0092-0.026) | 0.015(0.0084-0.025) |
| 0.1 | Skewed Continuous | 0.059(0.045-0.075) | 0.035(0.024-0.048) | 0.035(0.024-0.048) | 0.029(0.02-0.041) | 0.035(0.024-0.048) | 0.021(0.013-0.032) | 0.023(0.015-0.034) | 0.015(0.0084-0.025) | 0.018(0.011-0.028) |
| 0.1 | Dichotomous 50% | 0.033(0.023-0.046) | 0.006(0.0022-0.013) | 0.006(0.0022-0.013) | 0.009(0.0041-0.017) | 0.012(0.0062-0.021) | 0.009(0.0041-0.017) | 0.01(0.0048-0.018) | 0.008(0.0035-0.016) | 0.013(0.0069-0.022) |
| 0.1 | Dichotomous 25% | 0.049(0.036-0.064) | 0.018(0.011-0.028) | 0.02(0.012-0.031) | 0.024(0.015-0.036) | 0.023(0.015-0.034) | 0.007(0.0028-0.014) | 0.007(0.0028-0.014) | 0.011(0.0055-0.02) | 0.015(0.0084-0.025) |
| 0.2 | Normal | 0.033(0.023-0.046) | 0.017(0.0099-0.027) | 0.017(0.0099-0.027) | 0.017(0.0099-0.027) | 0.024(0.015-0.036) | 0.012(0.0062-0.021) | 0.011(0.0055-0.02) | 0.014(0.0077-0.023) | 0.018(0.011-0.028) |
| 0.2 | Skewed Continuous | 0.057(0.043-0.073) | 0.036(0.025-0.049) | 0.037(0.026-0.051) | 0.035(0.024-0.048) | 0.041(0.03-0.055) | 0.021(0.013-0.032) | 0.024(0.015-0.036) | 0.016(0.0092-0.026) | 0.019(0.011-0.03) |
| 0.2 | Dichotomous 50% | 0.034(0.024-0.047) | 0.005(0.0016-0.012) | 0.005(0.0016-0.012) | 0.009(0.0041-0.017) | 0.014(0.0077-0.023) | 0.011(0.0055-0.02) | 0.012(0.0062-0.021) | 0.012(0.0062-0.021) | 0.016(0.0092-0.026) |
| 0.2 | Dichotomous 25% | 0.055(0.042-0.071) | 0.012(0.0062-0.021) | 0.015(0.0084-0.025) | 0.02(0.012-0.031) | 0.021(0.013-0.032) | 0.008(0.0035-0.016) | 0.009(0.0041-0.017) | 0.017(0.0099-0.027) | 0.023(0.015-0.034) |
| 0.3 | Normal | 0.034(0.024-0.047) | 0.016(0.0092-0.026) | 0.017(0.0099-0.027) | 0.017(0.0099-0.027) | 0.024(0.015-0.036) | 0.014(0.0077-0.023) | 0.013(0.0069-0.022) | 0.013(0.0069-0.022) | 0.021(0.013-0.032) |
| 0.3 | Skewed Continuous | 0.056(0.043-0.072) | 0.03(0.02-0.043) | 0.03(0.02-0.043) | 0.028(0.019-0.04) | 0.038(0.027-0.052) | 0.019(0.011-0.03) | 0.02(0.012-0.031) | 0.017(0.0099-0.027) | 0.019(0.011-0.03) |
| 0.3 | Dichotomous 50% | 0.039(0.028-0.053) | 0.005(0.0016-0.012) | 0.005(0.0016-0.012) | 0.008(0.0035-0.016) | 0.015(0.0084-0.025) | 0.009(0.0041-0.017) | 0.01(0.0048-0.018) | 0.012(0.0062-0.021) | 0.016(0.0092-0.026) |
| 0.3 | Dichotomous 25% | 0.047(0.035-0.062) | 0.015(0.0084-0.025) | 0.016(0.0092-0.026) | 0.02(0.012-0.031) | 0.026(0.017-0.038) | 0.012(0.0062-0.021) | 0.012(0.0062-0.021) | 0.018(0.011-0.028) | 0.028(0.019-0.04) |
| 0.4 | Normal | 0.035(0.024-0.048) | 0.018(0.011-0.028) | 0.019(0.011-0.03) | 0.017(0.0099-0.027) | 0.025(0.016-0.037) | 0.015(0.0084-0.025) | 0.016(0.0092-0.026) | 0.016(0.0092-0.026) | 0.024(0.015-0.036) |
| 0.4 | Skewed Continuous | 0.052(0.039-0.068) | 0.029(0.02-0.041) | 0.027(0.018-0.039) | 0.027(0.018-0.039) | 0.037(0.026-0.051) | 0.02(0.012-0.031) | 0.023(0.015-0.034) | 0.022(0.014-0.033) | 0.022(0.014-0.033) |
| 0.4 | Dichotomous 50% | 0.044(0.032-0.059) | 0.007(0.0028-0.014) | 0.009(0.0041-0.017) | 0.011(0.0055-0.02) | 0.016(0.0092-0.026) | 0.008(0.0035-0.016) | 0.009(0.0041-0.017) | 0.01(0.0048-0.018) | 0.02(0.012-0.031) |
| 0.4 | Dichotomous 25% | 0.051(0.038-0.067) | 0.017(0.0099-0.027) | 0.018(0.011-0.028) | 0.024(0.015-0.036) | 0.029(0.02-0.041) | 0.009(0.0041-0.017) | 0.007(0.0028-0.014) | 0.015(0.0084-0.025) | 0.03(0.02-0.043) |
| 0.5 | Normal | 0.04(0.029-0.054) | 0.015(0.0084-0.025) | 0.014(0.0077-0.023) | 0.013(0.0069-0.022) | 0.021(0.013-0.032) | 0.012(0.0062-0.021) | 0.014(0.0077-0.023) | 0.015(0.0084-0.025) | 0.021(0.013-0.032) |
| 0.5 | Skewed Continuous | 0.058(0.044-0.074) | 0.031(0.021-0.044) | 0.031(0.021-0.044) | 0.035(0.024-0.048) | 0.038(0.027-0.052) | 0.019(0.011-0.03) | 0.02(0.012-0.031) | 0.025(0.016-0.037) | 0.021(0.013-0.032) |
| 0.5 | Dichotomous 50% | 0.042(0.03-0.056) | 0.008(0.0035-0.016) | 0.01(0.0048-0.018) | 0.012(0.0062-0.021) | 0.017(0.0099-0.027) | 0.007(0.0028-0.014) | 0.007(0.0028-0.014) | 0.009(0.0041-0.017) | 0.02(0.012-0.031) |
| 0.5 | Dichotomous 25% | 0.043(0.031-0.057) | 0.019(0.011-0.03) | 0.018(0.011-0.028) | 0.024(0.015-0.036) | 0.028(0.019-0.04) | 0.013(0.0069-0.022) | 0.01(0.0048-0.018) | 0.016(0.0092-0.026) | 0.031(0.021-0.044) |

1. MultiPC

| **Minimal**  **MAC** | **Phenotype** | **1st PC** | **MultiPC** | | | |
| --- | --- | --- | --- | --- | --- | --- |
|  |  |  | **99%** | **95%** | **90%** | **80%** |
| 0 | Normal | 0.031(0.021-0.044) | 0.053(0.04-0.069) | 0.051(0.038-0.067) | 0.05(0.037-0.065) | 0.046(0.034-0.061) |
| 0 | Skewed Continuous | 0.061(0.047-0.078) | 0.11(0.095-0.14) | 0.11(0.093-0.13) | 0.11(0.089-0.13) | 0.11(0.092-0.13) |
| 0 | Dichotomous 50% | 0.045(0.033-0.06) | 0.11(0.093-0.13) | 0.11(0.088-0.13) | 0.096(0.078-0.12) | 0.091(0.074-0.11) |
| 0 | Dichotomous 25% | 0.065(0.051-0.082) | 0.15(0.13-0.18) | 0.14(0.12-0.17) | 0.14(0.12-0.17) | 0.12(0.099-0.14) |
| 0.1 | Normal | 0.033(0.023-0.046) | 0.057(0.043-0.073) | 0.055(0.042-0.071) | 0.051(0.038-0.067) | 0.047(0.035-0.062) |
| 0.1 | Skewed Continuous | 0.059(0.045-0.075) | 0.11(0.088-0.13) | 0.098(0.08-0.12) | 0.093(0.076-0.11) | 0.097(0.079-0.12) |
| 0.1 | Dichotomous 50% | 0.044(0.032-0.059) | 0.11(0.094-0.13) | 0.11(0.089-0.13) | 0.098(0.08-0.12) | 0.089(0.072-0.11) |
| 0.1 | Dichotomous 25% | 0.065(0.051-0.082) | 0.17(0.14-0.19) | 0.15(0.13-0.18) | 0.15(0.13-0.18) | 0.12(0.1-0.15) |
| 0.2 | Normal | 0.033(0.023-0.046) | 0.054(0.041-0.07) | 0.051(0.038-0.067) | 0.045(0.033-0.06) | 0.043(0.031-0.057) |
| 0.2 | Skewed Continuous | 0.057(0.043-0.073) | 0.086(0.069-0.11) | 0.085(0.068-0.1) | 0.081(0.065-0.1) | 0.083(0.067-0.1) |
| 0.2 | Dichotomous 50% | 0.051(0.038-0.067) | 0.12(0.097-0.14) | 0.11(0.094-0.13) | 0.097(0.079-0.12) | 0.078(0.062-0.096) |
| 0.2 | Dichotomous 25% | 0.064(0.05-0.081) | 0.17(0.14-0.19) | 0.15(0.13-0.18) | 0.14(0.12-0.17) | 0.12(0.097-0.14) |
| 0.3 | Normal | 0.034(0.024-0.047) | 0.049(0.036-0.064) | 0.049(0.036-0.064) | 0.044(0.032-0.059) | 0.042(0.03-0.056) |
| 0.3 | Skewed Continuous | 0.056(0.043-0.072) | 0.081(0.065-0.1) | 0.076(0.06-0.094) | 0.075(0.059-0.093) | 0.074(0.059-0.092) |
| 0.3 | Dichotomous 50% | 0.052(0.039-0.068) | 0.11(0.091-0.13) | 0.11(0.088-0.13) | 0.089(0.072-0.11) | 0.071(0.056-0.089) |
| 0.3 | Dichotomous 25% | 0.062(0.048-0.079) | 0.16(0.13-0.18) | 0.14(0.12-0.17) | 0.13(0.11-0.15) | 0.11(0.09-0.13) |
| 0.4 | Normal | 0.035(0.024-0.048) | 0.051(0.038-0.067) | 0.053(0.04-0.069) | 0.048(0.036-0.063) | 0.049(0.036-0.064) |
| 0.4 | Skewed Continuous | 0.052(0.039-0.068) | 0.084(0.068-0.1) | 0.077(0.061-0.095) | 0.073(0.058-0.091) | 0.066(0.051-0.083) |
| 0.4 | Dichotomous 50% | 0.056(0.043-0.072) | 0.11(0.089-0.13) | 0.1(0.083-0.12) | 0.091(0.074-0.11) | 0.074(0.059-0.092) |
| 0.4 | Dichotomous 25% | 0.062(0.048-0.079) | 0.16(0.13-0.18) | 0.14(0.12-0.16) | 0.14(0.11-0.16) | 0.11(0.091-0.13) |
| 0.5 | Normal | 0.04(0.029-0.054) | 0.05(0.037-0.065) | 0.05(0.037-0.065) | 0.045(0.033-0.06) | 0.045(0.033-0.06) |
| 0.5 | Skewed Continuous | 0.058(0.044-0.074) | 0.083(0.067-0.1) | 0.075(0.059-0.093) | 0.067(0.052-0.084) | 0.061(0.047-0.078) |
| 0.5 | Dichotomous 50% | 0.054(0.041-0.07) | 0.099(0.081-0.12) | 0.092(0.075-0.11) | 0.084(0.068-0.1) | 0.072(0.057-0.09) |
| 0.5 | Dichotomous 25% | 0.058(0.044-0.074) | 0.14(0.12-0.16) | 0.12(0.1-0.15) | 0.12(0.1-0.14) | 0.1(0.083-0.12) |

*Minimal MAC: minimal maximum absolute pairwise correlation of a genomic region

*80%, 90%, 95%, 99%: minimal variance explained by PCs used.

*MetaPC: meta-analysis, MultiPC: multivariate regression.

**Supplementary** **Table 2. The Number of Genomic Regions Analyzed in Genome-wide False Positive Rates using 100 Discovery Cohort Subjects**

| Region Type | Using MAC_Residual_ | Number of Total Regions at Different Minimal MAC Cutoffs | | | | | |
| --- | --- | --- | --- | --- | --- | --- | --- |
|  |  | 0 | 0.1 | 0.2 | 0.3 | 0.4 | 0.5 |
| Genic | No | 44355 | 39281 | 26895 | 19799 | 15375 | 11998 |
| Genic | Yes | 44355 | 38549 | 24986 | 17424 | 12916 | 9558 |
| Intergenic | No | 9520 | 8300 | 5908 | 4597 | 3670 | 2866 |
| Intergenic | Yes | 9520 | 8074 | 5422 | 4018 | 3064 | 2377 |

* MAC_residual_: minimal maximum absolute pairwise correlation of a genomic region computed using residual M-values, where residual M-values are computed by adjusting for the same covariate set used in computing PCs of methylation residuals (e.g. age, sex, ancestry PCs, blood cell proportions).

**Supplementary Table 3. The Summary of True Positive Rates on Representative Regions**

1. MetaPC using Fisher’s Method

| **Region**  **ID** | **Phenotype** | **MetaPC1** | **True Positive Rates**  **using Fisher’s Method with Varying Minimal Variance Cutoffs** | | | |
| --- | --- | --- | --- | --- | --- | --- |
|  |  |  | **99%** | **95%** | **90%** | **80%** |
| 1 | CTS_PC1_ | 0.94(0.92-0.95) | 0.54(0.51-0.58) | 0.54(0.51-0.58) | 0.58(0.55-0.61) | 0.71(0.68-0.74) |
| 1 | CTS_PC2_ | 0.099(0.081-0.12) | 0.5(0.47-0.54) | 0.5(0.47-0.54) | 0.53(0.5-0.56) | 0.48(0.45-0.51) |
| 1 | CTS_PC1+PC2_ | 0.83(0.8-0.85) | 0.6(0.57-0.64) | 0.6(0.57-0.64) | 0.64(0.61-0.67) | 0.72(0.69-0.74) |
| 1 | DTS_PC1_ | 0.77(0.75-0.8) | 0.36(0.33-0.39) | 0.36(0.33-0.39) | 0.39(0.36-0.42) | 0.5(0.47-0.53) |
| 1 | DTS_PC2_ | 0.09(0.073-0.11) | 0.3(0.27-0.33) | 0.3(0.27-0.33) | 0.32(0.29-0.35) | 0.3(0.28-0.33) |
| 1 | DTS_PC1+PC2_ | 0.62(0.59-0.66) | 0.37(0.34-0.4) | 0.37(0.34-0.4) | 0.41(0.38-0.44) | 0.47(0.44-0.5) |
| 2 | CTS_PC1_ | 0.4(0.37-0.43) | 0.26(0.24-0.29) | 0.26(0.24-0.29) | 0.26(0.24-0.29) | 0.26(0.24-0.29) |
| 2 | CTS_PC2_ | 0.2(0.18-0.23) | 0.35(0.32-0.38) | 0.35(0.32-0.38) | 0.35(0.32-0.38) | 0.35(0.32-0.38) |
| 2 | CTS_PC1+PC2_ | 0.5(0.47-0.53) | 0.31(0.28-0.34) | 0.31(0.28-0.34) | 0.31(0.28-0.34) | 0.31(0.28-0.34) |
| 2 | DTS_PC1_ | 0.26(0.24-0.29) | 0.18(0.15-0.2) | 0.18(0.15-0.2) | 0.18(0.15-0.2) | 0.18(0.15-0.2) |
| 2 | DTS_PC2_ | 0.14(0.12-0.17) | 0.22(0.2-0.25) | 0.22(0.2-0.25) | 0.22(0.2-0.25) | 0.22(0.2-0.25) |
| 2 | DTS_PC1+PC2_ | 0.35(0.32-0.38) | 0.21(0.18-0.23) | 0.21(0.18-0.23) | 0.21(0.18-0.23) | 0.21(0.18-0.23) |
| 3 | CTS_PC1_ | 0.91(0.89-0.93) | 0.5(0.47-0.54) | 0.5(0.47-0.54) | 0.53(0.5-0.56) | 0.83(0.81-0.86) |
| 3 | CTS_PC2_ | 0.52(0.49-0.55) | 0.38(0.35-0.41) | 0.38(0.35-0.41) | 0.39(0.36-0.42) | 0.64(0.61-0.67) |
| 3 | CTS_PC1+PC2_ | 0.97(0.95-0.98) | 0.72(0.69-0.75) | 0.72(0.69-0.75) | 0.75(0.72-0.77) | 0.94(0.92-0.95) |
| 3 | DTS_PC1_ | 0.72(0.69-0.74) | 0.32(0.29-0.34) | 0.32(0.29-0.34) | 0.33(0.3-0.36) | 0.59(0.56-0.62) |
| 3 | DTS_PC2_ | 0.36(0.33-0.39) | 0.26(0.23-0.29) | 0.26(0.23-0.29) | 0.27(0.24-0.3) | 0.45(0.41-0.48) |
| 3 | DTS_PC1+PC2_ | 0.83(0.8-0.85) | 0.48(0.44-0.51) | 0.48(0.44-0.51) | 0.5(0.47-0.53) | 0.78(0.75-0.81) |
| 4 | CTS_PC1_ | 0.83(0.81-0.86) | 0.67(0.64-0.7) | 0.83(0.81-0.86) | 0.83(0.81-0.86) | 0.83(0.81-0.86) |
| 4 | CTS_PC2_ | 0.68(0.65-0.71) | 0.53(0.5-0.56) | 0.68(0.65-0.71) | 0.68(0.65-0.71) | 0.68(0.65-0.71) |
| 4 | CTS_PC1+PC2_ | 0.97(0.96-0.98) | 0.9(0.88-0.92) | 0.97(0.96-0.98) | 0.97(0.96-0.98) | 0.97(0.96-0.98) |
| 4 | DTS_PC1_ | 0.66(0.63-0.69) | 0.48(0.45-0.51) | 0.66(0.63-0.69) | 0.66(0.63-0.69) | 0.66(0.63-0.69) |
| 4 | DTS_PC2_ | 0.48(0.45-0.51) | 0.36(0.33-0.39) | 0.48(0.45-0.51) | 0.48(0.45-0.51) | 0.48(0.45-0.51) |
| 4 | DTS_PC1+PC2_ | 0.86(0.84-0.89) | 0.71(0.68-0.73) | 0.86(0.84-0.89) | 0.86(0.84-0.89) | 0.86(0.84-0.89) |
| 5 | CTS_PC1_ | 0.79(0.76-0.81) | 0.64(0.61-0.67) | 0.71(0.68-0.74) | 0.71(0.68-0.74) | 0.71(0.68-0.74) |
| 5 | CTS_PC2_ | 0.18(0.15-0.2) | 0.7(0.67-0.72) | 0.76(0.73-0.78) | 0.76(0.73-0.78) | 0.76(0.73-0.78) |
| 5 | CTS_PC1+PC2_ | 0.76(0.74-0.79) | 0.76(0.74-0.79) | 0.81(0.79-0.84) | 0.81(0.79-0.84) | 0.81(0.79-0.84) |
| 5 | DTS_PC1_ | 0.6(0.57-0.63) | 0.44(0.4-0.47) | 0.5(0.47-0.53) | 0.5(0.47-0.53) | 0.5(0.47-0.53) |
| 5 | DTS_PC2_ | 0.12(0.1-0.15) | 0.48(0.45-0.51) | 0.56(0.53-0.59) | 0.56(0.53-0.59) | 0.56(0.53-0.59) |
| 5 | DTS_PC1+PC2_ | 0.56(0.53-0.59) | 0.54(0.51-0.57) | 0.6(0.57-0.63) | 0.6(0.57-0.63) | 0.6(0.57-0.63) |
| 6 | CTS_PC1_ | 0.41(0.38-0.44) | 0.2(0.18-0.23) | 0.2(0.18-0.23) | 0.2(0.18-0.23) | 0.2(0.18-0.23) |
| 6 | CTS_PC2_ | 0.079(0.063-0.097) | 0.12(0.11-0.15) | 0.12(0.11-0.15) | 0.12(0.11-0.15) | 0.12(0.11-0.15) |
| 6 | CTS_PC1+PC2_ | 0.35(0.32-0.38) | 0.19(0.17-0.21) | 0.19(0.17-0.21) | 0.19(0.17-0.21) | 0.19(0.17-0.21) |
| 6 | DTS_PC1_ | 0.26(0.24-0.29) | 0.13(0.11-0.15) | 0.13(0.11-0.15) | 0.13(0.11-0.15) | 0.13(0.11-0.15) |
| 6 | DTS_PC2_ | 0.057(0.043-0.073) | 0.095(0.078-0.11) | 0.095(0.078-0.11) | 0.095(0.078-0.11) | 0.095(0.078-0.11) |
| 6 | DTS_PC1+PC2_ | 0.24(0.22-0.27) | 0.11(0.089-0.13) | 0.11(0.089-0.13) | 0.11(0.089-0.13) | 0.11(0.089-0.13) |
| 7 | CTS_PC1_ | 0.53(0.5-0.56) | 0.58(0.55-0.61) | 0.58(0.55-0.61) | 0.43(0.4-0.46) | 0.43(0.4-0.46) |
| 7 | CTS_PC2_ | 0.035(0.024-0.048) | 0.57(0.54-0.6) | 0.57(0.54-0.6) | 0.61(0.58-0.64) | 0.61(0.58-0.64) |
| 7 | CTS_PC1+PC2_ | 0.3(0.27-0.33) | 0.54(0.51-0.57) | 0.54(0.51-0.57) | 0.43(0.4-0.46) | 0.43(0.4-0.46) |
| 7 | DTS_PC1_ | 0.37(0.34-0.4) | 0.37(0.34-0.4) | 0.37(0.34-0.4) | 0.27(0.24-0.3) | 0.27(0.24-0.3) |
| 7 | DTS_PC2_ | 0.045(0.033-0.06) | 0.36(0.33-0.39) | 0.36(0.33-0.39) | 0.39(0.36-0.42) | 0.39(0.36-0.42) |
| 7 | DTS_PC1+PC2_ | 0.22(0.19-0.24) | 0.34(0.31-0.37) | 0.34(0.31-0.37) | 0.29(0.26-0.32) | 0.29(0.26-0.32) |
| 8 | CTS_PC1_ | 0.5(0.46-0.53) | 0.6(0.57-0.63) | 0.6(0.57-0.63) | 0.6(0.57-0.63) | 0.49(0.46-0.52) |
| 8 | CTS_PC2_ | 0.087(0.07-0.11) | 0.31(0.28-0.34) | 0.31(0.28-0.34) | 0.31(0.28-0.34) | 0.29(0.26-0.32) |
| 8 | CTS_PC1+PC2_ | 0.42(0.39-0.45) | 0.47(0.44-0.5) | 0.47(0.44-0.5) | 0.47(0.44-0.5) | 0.3(0.27-0.33) |
| 8 | DTS_PC1_ | 0.34(0.31-0.37) | 0.4(0.37-0.43) | 0.4(0.37-0.43) | 0.4(0.37-0.43) | 0.3(0.28-0.33) |
| 8 | DTS_PC2_ | 0.076(0.06-0.094) | 0.22(0.19-0.25) | 0.22(0.19-0.25) | 0.22(0.19-0.25) | 0.22(0.19-0.24) |
| 8 | DTS_PC1+PC2_ | 0.29(0.26-0.32) | 0.3(0.28-0.33) | 0.3(0.28-0.33) | 0.3(0.28-0.33) | 0.19(0.17-0.22) |

1. MetaPC using Stouffer’s Method

| **Region**  **ID** | **Phenotype** | **MetaPC1** | **True Positive Rates**  **using Stouffer’s Method with Varying Minimal Variance Cutoffs** | | | |
| --- | --- | --- | --- | --- | --- | --- |
|  |  |  | **99%** | **95%** | **90%** | **80%** |
| 1 | CTS_PC1_ | 0.94(0.92-0.95) | 0.26(0.23-0.29) | 0.26(0.23-0.29) | 0.3(0.27-0.33) | 0.42(0.39-0.45) |
| 1 | CTS_PC2_ | 0.099(0.081-0.12) | 0.38(0.35-0.41) | 0.38(0.35-0.41) | 0.41(0.38-0.44) | 0.4(0.37-0.43) |
| 1 | CTS_PC1+PC2_ | 0.83(0.8-0.85) | 0.42(0.39-0.46) | 0.42(0.39-0.46) | 0.46(0.43-0.5) | 0.55(0.52-0.58) |
| 1 | DTS_PC1_ | 0.77(0.75-0.8) | 0.21(0.19-0.24) | 0.21(0.19-0.24) | 0.22(0.2-0.25) | 0.31(0.28-0.34) |
| 1 | DTS_PC2_ | 0.09(0.073-0.11) | 0.24(0.22-0.27) | 0.24(0.22-0.27) | 0.26(0.23-0.29) | 0.26(0.23-0.29) |
| 1 | DTS_PC1+PC2_ | 0.62(0.59-0.66) | 0.28(0.26-0.31) | 0.28(0.26-0.31) | 0.3(0.27-0.33) | 0.35(0.32-0.38) |
| 2 | CTS_PC1_ | 0.4(0.37-0.43) | 0.22(0.2-0.25) | 0.22(0.2-0.25) | 0.22(0.2-0.25) | 0.22(0.2-0.25) |
| 2 | CTS_PC2_ | 0.2(0.18-0.23) | 0.27(0.24-0.3) | 0.27(0.24-0.3) | 0.27(0.24-0.3) | 0.27(0.24-0.3) |
| 2 | CTS_PC1+PC2_ | 0.5(0.47-0.53) | 0.22(0.2-0.25) | 0.22(0.2-0.25) | 0.22(0.2-0.25) | 0.22(0.2-0.25) |
| 2 | DTS_PC1_ | 0.26(0.24-0.29) | 0.14(0.12-0.16) | 0.14(0.12-0.16) | 0.14(0.12-0.16) | 0.14(0.12-0.16) |
| 2 | DTS_PC2_ | 0.14(0.12-0.17) | 0.18(0.16-0.2) | 0.18(0.16-0.2) | 0.18(0.16-0.2) | 0.18(0.16-0.2) |
| 2 | DTS_PC1+PC2_ | 0.35(0.32-0.38) | 0.16(0.14-0.19) | 0.16(0.14-0.19) | 0.16(0.14-0.19) | 0.16(0.14-0.19) |
| 3 | CTS_PC1_ | 0.91(0.89-0.93) | 0.26(0.23-0.29) | 0.26(0.23-0.29) | 0.26(0.23-0.29) | 0.68(0.65-0.71) |
| 3 | CTS_PC2_ | 0.52(0.49-0.55) | 0.28(0.25-0.31) | 0.28(0.25-0.31) | 0.28(0.25-0.31) | 0.62(0.59-0.65) |
| 3 | CTS_PC1+PC2_ | 0.97(0.95-0.98) | 0.36(0.33-0.39) | 0.36(0.33-0.39) | 0.39(0.36-0.42) | 0.87(0.85-0.89) |
| 3 | DTS_PC1_ | 0.72(0.69-0.74) | 0.19(0.16-0.21) | 0.19(0.16-0.21) | 0.19(0.16-0.21) | 0.48(0.45-0.51) |
| 3 | DTS_PC2_ | 0.36(0.33-0.39) | 0.2(0.17-0.22) | 0.2(0.17-0.22) | 0.2(0.17-0.23) | 0.44(0.41-0.47) |
| 3 | DTS_PC1+PC2_ | 0.83(0.8-0.85) | 0.27(0.25-0.3) | 0.27(0.25-0.3) | 0.3(0.27-0.33) | 0.68(0.65-0.71) |
| 4 | CTS_PC1_ | 0.83(0.81-0.86) | 0.47(0.44-0.5) | 0.83(0.81-0.86) | 0.83(0.81-0.86) | 0.83(0.81-0.86) |
| 4 | CTS_PC2_ | 0.68(0.65-0.71) | 0.39(0.36-0.42) | 0.68(0.65-0.71) | 0.68(0.65-0.71) | 0.68(0.65-0.71) |
| 4 | CTS_PC1+PC2_ | 0.97(0.96-0.98) | 0.69(0.66-0.72) | 0.97(0.96-0.98) | 0.97(0.96-0.98) | 0.97(0.96-0.98) |
| 4 | DTS_PC1_ | 0.66(0.63-0.69) | 0.34(0.31-0.37) | 0.66(0.63-0.69) | 0.66(0.63-0.69) | 0.66(0.63-0.69) |
| 4 | DTS_PC2_ | 0.48(0.45-0.51) | 0.29(0.26-0.32) | 0.48(0.45-0.51) | 0.48(0.45-0.51) | 0.48(0.45-0.51) |
| 4 | DTS_PC1+PC2_ | 0.86(0.84-0.89) | 0.5(0.47-0.53) | 0.86(0.84-0.89) | 0.86(0.84-0.89) | 0.86(0.84-0.89) |
| 5 | CTS_PC1_ | 0.79(0.76-0.81) | 0.5(0.47-0.54) | 0.6(0.56-0.63) | 0.6(0.56-0.63) | 0.6(0.56-0.63) |
| 5 | CTS_PC2_ | 0.18(0.15-0.2) | 0.56(0.53-0.59) | 0.7(0.67-0.72) | 0.7(0.67-0.72) | 0.7(0.67-0.72) |
| 5 | CTS_PC1+PC2_ | 0.76(0.74-0.79) | 0.67(0.64-0.7) | 0.79(0.76-0.81) | 0.79(0.76-0.81) | 0.79(0.76-0.81) |
| 5 | DTS_PC1_ | 0.6(0.57-0.63) | 0.33(0.3-0.36) | 0.41(0.38-0.44) | 0.41(0.38-0.44) | 0.41(0.38-0.44) |
| 5 | DTS_PC2_ | 0.12(0.1-0.15) | 0.38(0.35-0.41) | 0.5(0.47-0.53) | 0.5(0.47-0.53) | 0.5(0.47-0.53) |
| 5 | DTS_PC1+PC2_ | 0.56(0.53-0.59) | 0.47(0.44-0.5) | 0.58(0.55-0.62) | 0.58(0.55-0.62) | 0.58(0.55-0.62) |
| 6 | CTS_PC1_ | 0.41(0.38-0.44) | 0.17(0.15-0.19) | 0.17(0.15-0.19) | 0.17(0.15-0.19) | 0.17(0.15-0.19) |
| 6 | CTS_PC2_ | 0.079(0.063-0.097) | 0.11(0.088-0.13) | 0.11(0.088-0.13) | 0.11(0.088-0.13) | 0.11(0.088-0.13) |
| 6 | CTS_PC1+PC2_ | 0.35(0.32-0.38) | 0.15(0.13-0.17) | 0.15(0.13-0.17) | 0.15(0.13-0.17) | 0.15(0.13-0.17) |
| 6 | DTS_PC1_ | 0.26(0.24-0.29) | 0.11(0.095-0.14) | 0.11(0.095-0.14) | 0.11(0.095-0.14) | 0.11(0.095-0.14) |
| 6 | DTS_PC2_ | 0.057(0.043-0.073) | 0.075(0.059-0.093) | 0.075(0.059-0.093) | 0.075(0.059-0.093) | 0.075(0.059-0.093) |
| 6 | DTS_PC1+PC2_ | 0.24(0.22-0.27) | 0.1(0.082-0.12) | 0.1(0.082-0.12) | 0.1(0.082-0.12) | 0.1(0.082-0.12) |
| 7 | CTS_PC1_ | 0.53(0.5-0.56) | 0.48(0.45-0.51) | 0.48(0.45-0.51) | 0.35(0.32-0.38) | 0.35(0.32-0.38) |
| 7 | CTS_PC2_ | 0.035(0.024-0.048) | 0.47(0.43-0.5) | 0.47(0.43-0.5) | 0.52(0.49-0.55) | 0.52(0.49-0.55) |
| 7 | CTS_PC1+PC2_ | 0.3(0.27-0.33) | 0.48(0.45-0.52) | 0.48(0.45-0.52) | 0.39(0.36-0.42) | 0.39(0.36-0.42) |
| 7 | DTS_PC1_ | 0.37(0.34-0.4) | 0.31(0.28-0.34) | 0.31(0.28-0.34) | 0.22(0.2-0.25) | 0.22(0.2-0.25) |
| 7 | DTS_PC2_ | 0.045(0.033-0.06) | 0.28(0.25-0.31) | 0.28(0.25-0.31) | 0.32(0.29-0.35) | 0.32(0.29-0.35) |
| 7 | DTS_PC1+PC2_ | 0.22(0.19-0.24) | 0.33(0.3-0.36) | 0.33(0.3-0.36) | 0.27(0.24-0.3) | 0.27(0.24-0.3) |
| 8 | CTS_PC1_ | 0.5(0.46-0.53) | 0.55(0.52-0.58) | 0.55(0.52-0.58) | 0.55(0.52-0.58) | 0.45(0.42-0.49) |
| 8 | CTS_PC2_ | 0.087(0.07-0.11) | 0.28(0.26-0.31) | 0.28(0.26-0.31) | 0.28(0.26-0.31) | 0.27(0.24-0.3) |
| 8 | CTS_PC1+PC2_ | 0.42(0.39-0.45) | 0.38(0.35-0.41) | 0.38(0.35-0.41) | 0.38(0.35-0.41) | 0.22(0.2-0.25) |
| 8 | DTS_PC1_ | 0.34(0.31-0.37) | 0.37(0.34-0.4) | 0.37(0.34-0.4) | 0.37(0.34-0.4) | 0.27(0.24-0.29) |
| 8 | DTS_PC2_ | 0.076(0.06-0.094) | 0.2(0.18-0.23) | 0.2(0.18-0.23) | 0.2(0.18-0.23) | 0.2(0.17-0.23) |
| 8 | DTS_PC1+PC2_ | 0.29(0.26-0.32) | 0.24(0.21-0.27) | 0.24(0.21-0.27) | 0.24(0.21-0.27) | 0.16(0.13-0.18) |

1. MultiPC

| **Region**  **ID** | **Phenotype** | **MultiPC1** | **True Positive Rates**  **with Varying Minimal Variance Cutoffs** | | | |
| --- | --- | --- | --- | --- | --- | --- |
|  |  |  | **99%** | **95%** | **90%** | **80%** |
| 1 | CTS_PC1_ | 0.94(0.92-0.95) | 0.62(0.59-0.65) | 0.62(0.59-0.65) | 0.65(0.62-0.68) | 0.76(0.73-0.79) |
| 1 | CTS_PC2_ | 0.099(0.081-0.12) | 0.52(0.49-0.55) | 0.52(0.49-0.55) | 0.54(0.51-0.57) | 0.48(0.45-0.51) |
| 1 | CTS_PC1+PC2_ | 0.83(0.8-0.85) | 0.64(0.61-0.67) | 0.64(0.61-0.67) | 0.66(0.63-0.69) | 0.72(0.69-0.75) |
| 1 | DTS_PC1_ | 0.77(0.75-0.8) | 0.43(0.4-0.47) | 0.43(0.4-0.47) | 0.45(0.42-0.48) | 0.54(0.51-0.57) |
| 1 | DTS_PC2_ | 0.09(0.073-0.11) | 0.33(0.3-0.36) | 0.33(0.3-0.36) | 0.34(0.31-0.37) | 0.32(0.29-0.35) |
| 1 | DTS_PC1+PC2_ | 0.63(0.6-0.66) | 0.42(0.39-0.45) | 0.42(0.39-0.45) | 0.44(0.41-0.47) | 0.50(0.47-0.53) |
| 2 | CTS_PC1_ | 0.4(0.37-0.43) | 0.28(0.25-0.31) | 0.28(0.25-0.31) | 0.28(0.25-0.31) | 0.28(0.25-0.31) |
| 2 | CTS_PC2_ | 0.2(0.18-0.23) | 0.36(0.33-0.39) | 0.36(0.33-0.39) | 0.36(0.33-0.39) | 0.36(0.33-0.39) |
| 2 | CTS_PC1+PC2_ | 0.5(0.47-0.53) | 0.32(0.3-0.35) | 0.32(0.3-0.35) | 0.32(0.3-0.35) | 0.32(0.3-0.35) |
| 2 | DTS_PC1_ | 0.27(0.24-0.3) | 0.19(0.17-0.21) | 0.19(0.17-0.21) | 0.19(0.17-0.21) | 0.19(0.17-0.21) |
| 2 | DTS_PC2_ | 0.14(0.12-0.17) | 0.24(0.21-0.27) | 0.24(0.21-0.27) | 0.24(0.21-0.27) | 0.24(0.21-0.27) |
| 2 | DTS_PC1+PC2_ | 0.35(0.32-0.38) | 0.23(0.21-0.26) | 0.23(0.21-0.26) | 0.23(0.21-0.26) | 0.23(0.21-0.26) |
| 3 | CTS_PC1_ | 0.91(0.89-0.93) | 0.56(0.53-0.59) | 0.56(0.53-0.59) | 0.58(0.55-0.61) | 0.84(0.82-0.87) |
| 3 | CTS_PC2_ | 0.52(0.49-0.55) | 0.41(0.38-0.44) | 0.41(0.38-0.44) | 0.41(0.38-0.45) | 0.63(0.6-0.66) |
| 3 | CTS_PC1+PC2_ | 0.97(0.95-0.98) | 0.78(0.76-0.81) | 0.78(0.76-0.81) | 0.79(0.77-0.82) | 0.94(0.93-0.96) |
| 3 | DTS_PC1_ | 0.72(0.69-0.75) | 0.36(0.33-0.39) | 0.36(0.33-0.39) | 0.38(0.35-0.41) | 0.61(0.58-0.64) |
| 3 | DTS_PC2_ | 0.36(0.33-0.39) | 0.29(0.26-0.32) | 0.29(0.26-0.32) | 0.29(0.26-0.32) | 0.45(0.42-0.48) |
| 3 | DTS_PC1+PC2_ | 0.83(0.8-0.85) | 0.55(0.52-0.58) | 0.55(0.52-0.58) | 0.56(0.52-0.59) | 0.79(0.77-0.82) |
| 4 | CTS_PC1_ | 0.83(0.81-0.86) | 0.69(0.66-0.72) | 0.83(0.81-0.86) | 0.83(0.81-0.86) | 0.83(0.81-0.86) |
| 4 | CTS_PC2_ | 0.68(0.65-0.71) | 0.55(0.52-0.58) | 0.68(0.65-0.71) | 0.68(0.65-0.71) | 0.68(0.65-0.71) |
| 4 | CTS_PC1+PC2_ | 0.97(0.96-0.98) | 0.92(0.9-0.93) | 0.97(0.96-0.98) | 0.97(0.96-0.98) | 0.97(0.96-0.98) |
| 4 | DTS_PC1_ | 0.66(0.63-0.69) | 0.5(0.47-0.53) | 0.66(0.63-0.69) | 0.66(0.63-0.69) | 0.66(0.63-0.69) |
| 4 | DTS_PC2_ | 0.48(0.45-0.51) | 0.38(0.35-0.41) | 0.48(0.45-0.51) | 0.48(0.45-0.51) | 0.48(0.45-0.51) |
| 4 | DTS_PC1+PC2_ | 0.87(0.84-0.89) | 0.74(0.71-0.76) | 0.87(0.84-0.89) | 0.87(0.84-0.89) | 0.87(0.84-0.89) |
| 5 | CTS_PC1_ | 0.79(0.76-0.81) | 0.66(0.63-0.69) | 0.72(0.69-0.75) | 0.72(0.69-0.75) | 0.72(0.69-0.75) |
| 5 | CTS_PC2_ | 0.18(0.15-0.2) | 0.71(0.68-0.74) | 0.77(0.74-0.79) | 0.77(0.74-0.79) | 0.77(0.74-0.79) |
| 5 | CTS_PC1+PC2_ | 0.76(0.74-0.79) | 0.77(0.74-0.79) | 0.81(0.79-0.84) | 0.81(0.79-0.84) | 0.81(0.79-0.84) |
| 5 | DTS_PC1_ | 0.6(0.57-0.63) | 0.46(0.43-0.5) | 0.5(0.47-0.54) | 0.5(0.47-0.54) | 0.5(0.47-0.54) |
| 5 | DTS_PC2_ | 0.12(0.11-0.15) | 0.5(0.47-0.53) | 0.56(0.52-0.59) | 0.56(0.52-0.59) | 0.56(0.52-0.59) |
| 5 | DTS_PC1+PC2_ | 0.56(0.53-0.59) | 0.55(0.51-0.58) | 0.6(0.56-0.63) | 0.6(0.56-0.63) | 0.6(0.56-0.63) |
| 6 | CTS_PC1_ | 0.41(0.38-0.44) | 0.22(0.2-0.25) | 0.22(0.2-0.25) | 0.22(0.2-0.25) | 0.22(0.2-0.25) |
| 6 | CTS_PC2_ | 0.079(0.063-0.097) | 0.14(0.12-0.16) | 0.14(0.12-0.16) | 0.14(0.12-0.16) | 0.14(0.12-0.16) |
| 6 | CTS_PC1+PC2_ | 0.35(0.32-0.38) | 0.19(0.17-0.22) | 0.19(0.17-0.22) | 0.19(0.17-0.22) | 0.19(0.17-0.22) |
| 6 | DTS_PC1_ | 0.27(0.24-0.29) | 0.14(0.12-0.16) | 0.14(0.12-0.16) | 0.14(0.12-0.16) | 0.14(0.12-0.16) |
| 6 | DTS_PC2_ | 0.059(0.045-0.075) | 0.1(0.082-0.12) | 0.1(0.082-0.12) | 0.1(0.082-0.12) | 0.1(0.082-0.12) |
| 6 | DTS_PC1+PC2_ | 0.24(0.22-0.27) | 0.13(0.11-0.15) | 0.13(0.11-0.15) | 0.13(0.11-0.15) | 0.13(0.11-0.15) |
| 7 | CTS_PC1_ | 0.53(0.5-0.56) | 0.59(0.56-0.62) | 0.59(0.56-0.62) | 0.43(0.4-0.46) | 0.43(0.4-0.46) |
| 7 | CTS_PC2_ | 0.035(0.024-0.048) | 0.58(0.55-0.61) | 0.58(0.55-0.61) | 0.61(0.58-0.64) | 0.61(0.58-0.64) |
| 7 | CTS_PC1+PC2_ | 0.3(0.27-0.33) | 0.54(0.51-0.57) | 0.54(0.51-0.57) | 0.43(0.4-0.46) | 0.43(0.4-0.46) |
| 7 | DTS_PC1_ | 0.37(0.34-0.4) | 0.39(0.36-0.42) | 0.39(0.36-0.42) | 0.29(0.26-0.32) | 0.29(0.26-0.32) |
| 7 | DTS_PC2_ | 0.045(0.033-0.06) | 0.39(0.36-0.42) | 0.39(0.36-0.42) | 0.42(0.39-0.45) | 0.42(0.39-0.45) |
| 7 | DTS_PC1+PC2_ | 0.22(0.19-0.24) | 0.36(0.33-0.39) | 0.36(0.33-0.39) | 0.29(0.26-0.32) | 0.29(0.26-0.32) |
| 8 | CTS_PC1_ | 0.5(0.46-0.53) | 0.6(0.57-0.63) | 0.6(0.57-0.63) | 0.6(0.57-0.63) | 0.48(0.45-0.51) |
| 8 | CTS_PC2_ | 0.087(0.07-0.11) | 0.32(0.3-0.35) | 0.32(0.3-0.35) | 0.32(0.3-0.35) | 0.29(0.26-0.32) |
| 8 | CTS_PC1+PC2_ | 0.42(0.39-0.45) | 0.48(0.45-0.51) | 0.48(0.45-0.51) | 0.48(0.45-0.51) | 0.31(0.28-0.34) |
| 8 | DTS_PC1_ | 0.34(0.31-0.37) | 0.41(0.38-0.44) | 0.41(0.38-0.44) | 0.41(0.38-0.44) | 0.31(0.28-0.34) |
| 8 | DTS_PC2_ | 0.076(0.06-0.094) | 0.22(0.2-0.25) | 0.22(0.2-0.25) | 0.22(0.2-0.25) | 0.21(0.19-0.24) |
| 8 | DTS_PC1+PC2_ | 0.29(0.26-0.32) | 0.31(0.28-0.34) | 0.31(0.28-0.34) | 0.31(0.28-0.34) | 0.2(0.18-0.23) |

1. coMethDMR

| **Region ID** | **CoMethDMR Region** | **Phenotype** | **True Positive Rates** |
| --- | --- | --- | --- |
| 1a | Chr6:30038754-30038882 | CTS_PC1_ | 0.12 (0.098-0.14) |
| 1a | Chr6:30038754-30038882 | CTS_PC2_ | 0.11 (0.09-0.13) |
| 1a | Chr6:30038754-30038882 | CTS_PC1+PC2_ | 0.6 (0.57-0.63) |
| 1a | Chr6:30038754-30038882 | DTS_PC1_ | 0.44 (0.41-0.47) |
| 1a | Chr6:30038754-30038882 | DTS_PC2_ | 0.094 (0.077-0.11) |
| 1a | Chr6:30038754-30038882 | DTS_PC1+PC2_ | 0.43 (0.4-0.46) |
| 1b | Chr6:30038922-30039206 | CTS_PC1_ | 0.052 (0.039-0.068) |
| 1b | Chr6:30038922-30039206 | CTS_PC2_ | 0.063 (0.049-0.08) |
| 1b | Chr6:30038922-30039206 | CTS_PC1+PC2_ | 0.64 (0.61-0.67) |
| 1b | Chr6:30038922-30039206 | DTS_PC1_ | 0.64 (0.61-0.67) |
| 1b | Chr6:30038922-30039206 | DTS_PC2_ | 0.076 (0.06-0.094) |
| 1b | Chr6:30038922-30039206 | DTS_PC1+PC2_ | 0.51 (0.48-0.54) |
| 1c | Chr6:30039374-30039600 | CTS_PC1_ | 0.021 (0.013-0.032) |
| 1c | Chr6:30039374-30039600 | CTS_PC2_ | 0.034 (0.024-0.047) |
| 1c | Chr6:30039374-30039600 | CTS_PC1+PC2_ | 0.66 (0.63-0.69) |
| 1c | Chr6:30039374-30039600 | DTS_PC1_ | 0.68 (0.65-0.7) |
| 1c | Chr6:30039374-30039600 | DTS_PC2_ | 0.063 (0.049-0.08) |
| 1c | Chr6:30039374-30039600 | DTS_PC1+PC2_ | 0.52 (0.48-0.55) |
| 1* | Chr6:30038712-30039600 | CTS_PC1_ | 0.17 (0.15, 0.20) |
| 1* | Chr6:30038712-30039600 | CTS_PC2_ | 0.15 (0.13, 0.17) |
| 1* | Chr6:30038712-30039600 | CTS_PC1+PC2_ | 0.84 (0.81, 0.86) |
| 1* | Chr6:30038712-30039600 | DTS_PC1_ | 0.78 (0.75, 0.81) |
| 1* | Chr6:30038712-30039600 | DTS_PC2_ | 0.14 (0.11, 0.16) |
| 1* | Chr6:30038712-30039600 | DTS_PC1+PC2_ | 0.66 (0.63, 0.69) |
| 3 | Chr7:27183133-27184737 | CTS_PC1_ | 0.034 (0.024-0.047) |
| 3 | Chr7:27183133-27184737 | CTS_PC2_ | 0.10 (0.082-0.12) |
| 3 | Chr7:27183133-27184737 | CTS_PC1+PC2_ | 0.69 (0.66-0.72) |
| 3 | Chr7:27183133-27184737 | DTS_PC1_ | 0.59 (0.56-0.62) |
| 3 | Chr7:27183133-27184737 | DTS_PC2_ | 0.28 (0.25-0.31) |
| 3 | Chr7:27183133-27184737 | DTS_PC1+PC2_ | 0.67 (0.64-0.7) |
| 4 | Chr1:248100585-248100614 | CTS_PC1_ | 0.35 (0.32-0.38) |
| 4 | Chr1:248100585-248100614 | CTS_PC2_ | 0.44 (0.41-0.47) |
| 4 | Chr1:248100585-248100614 | CTS_PC1+PC2_ | 0.92 (0.9-0.93) |
| 4 | Chr1:248100585-248100614 | DTS_PC1_ | 0.61 (0.58-0.64) |
| 4 | Chr1:248100585-248100614 | DTS_PC2_ | 0.44 (0.41-0.48) |
| 4 | Chr1:248100585-248100614 | DTS_PC1+PC2_ | 0.78 (0.76-0.81) |

* Region ID 1-8 corresponds to region names listed in our pre-defined regions as: 1 - Chr6:30038712-30039600, 2 - Chr6:31125920-31126373, 3 -Chr7:27183133-27184737, 4 - Chr1:248100585-248100614, 5 - Chr19:8117875-8117966, 6 - Chr10:8095121-8096372, 7 - Chr16:67312928-67313043 and 8 - Chr15:72104228-72104417. Note that coMethDMR splits region 1 into 3 sub-regions for analysis. We have denoted these region 1a, 1b, and 1c. Region 1* denotes the rate of a significant association in any of the 3 sub-regions.

**Supplementary Table 4. The Number of Genome-wide Significant CpG Sites in EWASs**

| **Cohort** | **Phenotype** | **# Subjects** | **# Genome-wide Significant**  **CpG Sites** |
| --- | --- | --- | --- |
| Discovery | Age | 528 | 142347 |
|  | Sex | 528 | 10517 |
|  | Smoking (0/1) | 400 | 2641 |
| Replication | Age | 647 | 291426 |
|  | Sex | 647 | 106859 |
|  | Smoking (0,1,2) | 461 | 450 |

**Supplementary Table 5. Summary of DMRs with any Genome-wide Significant Locus in EWAS**

| **Cohort** | **Phenotype** | **High correlation Regions**  **(Analyzed by Both Methods)** | | | | | | **Moderate correlation Regions**  **(Analyzed by DMR_PC_ Only)** | |
| --- | --- | --- | --- | --- | --- | --- | --- | --- | --- |
|  |  | **# DMRs** | | | **% DMRs**  **with EWAS Hits** | | | **# DMRs** | **% DMRs**  **with EWAS Hits** |
|  |  | **DMR_PC_** | **coMethDMR** | **Both** | **DMR_PC_** | **coMethDMR** | **Both** | **DMR_PC_** | **DMR_PC_** |
| Discovery | Age | 4252 | 2834 | 2644 | 98.64 | 98.98 | 100 | 4709 | 98.96 |
|  | Sex | 675 | 580 | 394 | 90.22 | 82.41 | 96.45 | 425 | 85.41 |
|  | Smoking  (0/1) | 21 | 45 | 9 | 95.24 | 68.89 | 100 | 21 | 90.48 |
| Replication | Age | 8812 | 5803 | 5683 | 99.14 | 99.81 | 100 | 9014 | 99.40 |
|  | Sex | 3419 | 2398 | 1818 | 97.98 | 96.50 | 99.89 | 2863 | 98.08 |
|  | Smoking  (0/1/2) | 14 | 6 | 3 | 100 | 66.67 | 100 | 3 | 100 |

*Under coMethDMR, raw regions refer to geonic regions from the original output from coMethDMR including those subregions, regions refer to geonic regions with subregions combined.

**Supplementary Figure 1. The Genome-wide False Positive Rates on Intergenic Regions using 100 Discovery Cohort Subjects**

*Genome-wide FP: genome-wide false positive rate.

*Minimal MAC: minimal maximum absolute pairwise correlation of a genomic region

*80%, 90%, 95%, 99%: minimal variance explained by PCs used.

*MetaPC: meta-analysis using multiple PCs, MultiPC: multivariate regression using multiple PCs.

*MetaPC1: meta-analysis using 1^st^ PC only, MultiPC1: multivariate regression using 1^st^ PC.

**Supplementary Figure 2. The True Positive Rates for Continuous Signals on Representative Regions**

1. Region 4

1. Region 5

1. Region 6

1. Region 7

1. Region 8

*80%, 90%, 95%, 99%: minimal variance explained by PCs used.

*MetaPC: meta-analysis using multiple PCs, MultiPC: multivariate regression using multiple PCs.

*MetaPC1: meta-analysis using 1^st^ PC only, MultiPC1: multivariate regression using 1^st^ PC only.

*PC1, PC2, PC1+PC2: true positive signals simulated associated with PC1, PC2, and PC1+PC2.
